# Supplementary material for: From Forensics to Clinical Research: Expanding the Variant Calling Pipeline for the Precision ID mtDNA Whole Genome Panel
Source: Int J Mol Sci. 2021 Nov 6;22(21):12031. doi: 10.3390/ijms222112031 (PMC8584537; doi:10.3390/ijms222112031)
Supplement: Supplementary file 1 [file ijms-22-12031-s001.zip › ijms-1433021-supplementary.pdf]

Article

From Forensics to Clinical Research: Expanding the Variant Calling Pipeline for the Precision ID mtDNA Whole Genome Panel

Filipe Cortes-Figueiredo [1,2], Filipa S. Carvalho [1], Ana Catarina Fonseca [3,4], Friedemann Paul [2,5], José M. Ferro [3,4], Sebastian Schönherr [6], Hansi Weissensteiner [6,*] and Vanessa A. Morais [1,*]

[1] VMorais Lab – Mitochondria Biology & Neurodegeneration, Instituto de Medicina Molecular João Lobo Antunes, Faculdade de Medicina, Universidade de Lisboa, Lisbon 1649-028, Portugal; filipe.figueiredo@medicina.ulisboa.pt (F.C.F.); filcarvalho.uc@gmail.com (F.S.C.)

[2] NeuroCure Clinical Research Center, Charité – Universitätsmedizin Berlin, Berlin 10117, Germany; friedemann.paul@charite.de (F.P.)

[3] José Ferro Lab – Clinical Research in Non-communicable Neurological Diseases, Instituto de Medicina Molecular João Lobo Antunes, Faculdade de Medicina, Universidade de Lisboa, Lisbon 1649-028, Portugal; acfonseca@medicina.ulisboa.pt (A.C.F.); jmferro@medicina.ulisboa.pt (J.M.F.)

[4] Serviço de Neurologia, Hospital de Santa Maria, Centro Hospitalar Universitário Lisboa Norte, Lisbon 1649-035, Portugal

[5] Experimental and Clinical Research Center, Charité – Universitätsmedizin Berlin and Max Delbrück Center for Molecular Medicine, Berlin 13125, Germany

[6] Institute of Genetic Epidemiology, Department of Genetics and Pharmacology, Medical University of Innsbruck, Innsbruck 6020, Austria; sebastian.schoenherr@i-med.ac.at (S.S.)

* Correspondence: hansi.weissensteiner@i-med.ac.at (H.W.); vmorais@medicina.ulisboa.pt (V.A.M.); Tel.: +43-0512-9003-70560 (H.W.); Tel.: +351-217-999-573 (V.A.M.)

SUPPLEMENTARY MATERIAL

Contents

Tables

Table S1 - Mixture analysis: Variant classification methodology 3
Table S2 - Optimizing the approach to NUMT removal. Paired t-test with FDR 9
Table S3 - Optimizing the heteroplasmy threshold in mutserve, in datasets processed with RtN! before merging 10
Table S4 - Primary analysis: Overall look 11
Table S5 - Ion Torrent sequencing: Overall look on differences between PCP and TSS 12
Table S6 - Primary analysis: Distribution of Grade A/B variants, according to the VCM chosen. McNemar's test with FDR 15
Table S7 - Primary analysis: Paired difference between the observed VL in Ion Torrent and the expected VL in Grade A/B variants with other sequencing methods, according to the VCM chosen. Bootstrap with FDR 15
Table S8 - Primary analysis: Paired difference between the proportion of "Novel variants", depending on the VCM chosen. Bootstrap with FDR 15
Table S9 - Mixture analysis: Distribution of Grade A/B variants, according to the VCM chosen. McNemar's test with FDR 16
Table S10 - Mixture analysis: Paired difference between the observed VL and the expected VL (internal and external) in Grade A/B variants, according to the VCM chosen. Paired t-test with FDR 16
Table S11 - Mixture analysis: Paired relative difference between the observed VL and the expected VL (internal and external) in Grade A/B variants, according to the VCM chosen. Paired t-test with FDR 19
Table S12 - Mixture analysis: Paired difference between correlation indicators (internal and external) in Grade A/B variants, depending on the VCM chosen. Paired t-test with FDR 21
Table S13 - Mixture analysis: Paired difference between the proportion of "Novel variants", depending on the VCM chosen. Paired t-test with FDR 23
Table S14 - Mixture analysis: Paired difference between TSS and PCP VL for "Novel variants", depending on the VCM chosen. Paired t-test with FDR 23
Table S15 - Mixture analysis: Relative burden of different macro variants in different mtDNA regions, depending on the VCM chosen. All regions 25
Table S16 - Mixture analysis: Relative burden of different macro variants in different mtDNA regions, depending on the VCM chosen. Macro regions only 31
Table S17 - Mixture analysis: Paired comparison of mean normalized coverage per sample in different macro variants, depending on the VCM chosen. Pairwise t-test with FDR 34
Table S18 - Mixture analysis: Paired comparison of mean coverage ratios per sample in different macro variants, depending on the VCM chosen. Pairwise t-test with FDR 35
Table S19 - Mixture analysis: Paired comparison of NUMT rates per sample in different macro variants, depending on the VCM chosen. Pairwise t-test with FDR 36
Table S20 - Mixture analysis: Paired comparison of mean prevalence of LCR variants per sample in different macro variants, depending on the VCM chosen. Pairwise t-test with FDR 37
Table S21 - Mixture analysis: Paired comparison of mean distance to the amplicon's edge per sample in different macro variants, depending on the VCM chosen. Pairwise t-test with FDR 38
Table S22 - Performance analysis: Paired difference between different performance indicators for Grade A/B variants, depending on the VCM chosen. Bootstrap with FDR for the primary analysis and paired t-test with FDR for the mixture analysis 39
Table S23 - Primary analysis: Distribution of variants with freebayes and varscan 41
Table S24 - Mixture analysis: Distribution of variants with freebayes and varscan 43
Table S25 - Ion Torrent sequencing of an independent set of 50 clinical samples: Overall look on differences between PCP and TSS 47
Table S26 - Ion Torrent sequencing of an independent set of 50 clinical samples: Discordant variants between PCP and TSS 49

Figures

Figure S1 - Optimization of the bioinformatic processing. 8
Figure S2 - Mixture analysis: Overall look with PCP, in mutserve. 13
Figure S3 - Distribution of variant levels with mutserve, per pipeline. 14
Figure S4 - Differences between different variant classifications, per pipeline. 24
Figure S5 - Ion Torrent sequencing of an independent set of 50 clinical samples: Linear regression model between the variant level in PCP and the variant level in TSS, for variants found in both PCP and TSS. 54

List of abbreviations
adj - adjusted

CI - confidence interval

FDR - false discovery rate

HVS - hypervariable segment

LCR - low complexity region

Max - maximum

Min - minimum

mtDNA - mitochondrial DNA

N - number

NUMT - nuclear insertion of mitochondrial DNA

PCP - PrecisionCallerPipeline

Poly-C - poly-C stretch

TSS - Ion Torrent Suite™ Software

VCM - variant calling method

VL - variant level

Table S1 - Mixture analysis: Variant classification methodology

Minor component: primary analysis VL	Minor component: primary analysis variant class	Major component: primary analysis VL	Major component: primary analysis variant class	Mixture: VL	Mixture: micro	Mixture: meso	Mixture: macro
0	-	> 0	Grade A variant found	> 0	Major Grade A variant found (Ion Torrent and mixture)	Found variants	Found variants
0	-	> 0	Grade B variant found	> 0	Major Grade B variant found (Ion Torrent and mixture)	Found variants	Found variants
0	-	> 0	Grade C variant found	> 0	Major Grade C variant found (Ion Torrent and mixture)	Found variants	Found variants
> 0	Grade A variant found	0	-	> 0	Minor Grade A variant found (Ion Torrent and mixture)	Found variants	Found variants
> 0	Grade B variant found	0	-	> 0	Minor Grade B variant found (Ion Torrent and mixture)	Found variants	Found variants
> 0	Grade C variant found	0	-	> 0	Minor Grade C variant found (Ion Torrent and mixture)	Found variants	Found variants
> 0	Grade A variant found	> 0	Grade A variant found	> 0	Shared Grade A variant found (Ion Torrent and mixture)	Found variants	Found variants
> 0	Grade A variant found	> 0	Grade B variant found	> 0	Shared Grade B variant found (Ion Torrent and mixture)	Found variants	Found variants
> 0	Grade B variant found	> 0	Grade A variant found	> 0	Shared Grade B variant found (Ion Torrent and mixture)	Found variants	Found variants
> 0	Grade B variant found	> 0	Grade B variant found	> 0	Shared Grade B variant found (Ion Torrent and mixture)	Found variants	Found variants
> 0	Grade A variant found	> 0	Grade C variant found	> 0	Shared Grade C variant found (Ion Torrent and mixture)	Found variants	Found variants
> 0	Grade B variant found	> 0	Grade C variant found	> 0	Shared Grade C variant found (Ion Torrent and mixture)	Found variants	Found variants
> 0	Grade C variant found	> 0	Grade A variant found	> 0	Shared Grade C variant found (Ion Torrent and mixture)	Found variants	Found variants
> 0	Grade C variant found	> 0	Grade B variant found	> 0	Shared Grade C variant found (Ion Torrent and mixture)	Found variants	Found variants
> 0	Grade C variant found	> 0	Grade C variant found	> 0	Shared Grade C variant found (Ion Torrent and mixture)	Found variants	Found variants
0	-	0	Grade A variant lost	> 0	Major Grade A variant resurfaced (mixture)	Mixture found variants	Found variants
0	-	0	Grade B variant lost	> 0	Major Grade B variant resurfaced (mixture)	Mixture found variants	Found variants
0	-	0	Grade C variant lost	> 0	Major Grade C variant resurfaced (mixture)	Mixture found variants	Found variants
0	Grade A variant lost	0	-	> 0	Minor Grade A variant resurfaced (mixture)	Mixture found variants	Found variants
0	Grade B variant lost	0	-	> 0	Minor Grade B variant resurfaced (mixture)	Mixture found variants	Found variants
0	Grade C variant lost	0	-	> 0	Minor Grade C variant resurfaced (mixture)	Mixture found variants	Found variants
0	Grade A variant lost	0	Grade A variant lost	> 0	Shared Grade A variant resurfaced (mixture)	Mixture found variants	Found variants
0	Grade A variant lost	0	Grade B variant lost	> 0	Shared Grade B variant resurfaced (mixture)	Mixture found variants	Found variants
0	Grade B variant lost	0	Grade A variant lost	> 0	Shared Grade B variant resurfaced (mixture)	Mixture found variants	Found variants
0	Grade B variant lost	0	Grade B variant lost	> 0	Shared Grade B variant resurfaced (mixture)	Mixture found variants	Found variants
0	Grade A variant lost	0	Grade C variant lost	> 0	Shared Grade C variant resurfaced (mixture)	Mixture found variants	Found variants
0	Grade B variant lost	0	Grade C variant lost	> 0	Shared Grade C variant resurfaced (mixture)	Mixture found variants	Found variants
0	Grade C variant lost	0	Grade A variant lost	> 0	Shared Grade C variant resurfaced (mixture)	Mixture found variants	Found variants
0	Grade C variant lost	0	Grade B variant lost	> 0	Shared Grade C variant resurfaced (mixture)	Mixture found variants	Found variants
0	Grade C variant lost	0	Grade C variant lost	> 0	Shared Grade C variant resurfaced (mixture)	Mixture found variants	Found variants

Minor component: primary analysis VL	Minor component: primary analysis variant class	Major component: primary analysis VL	Major component: primary analysis variant class	Mixture: VL	Mixture: micro	Mixture: meso	Mixture: macro
0	-	> 0	Grade A variant found	0	Major Grade A variant lost (mixture)	Mixture lost variants	Lost variants
0	-	> 0	Grade B variant found	0	Major Grade B variant lost (mixture)	Mixture lost variants	Lost variants
0	-	> 0	Grade C variant found	0	Major Grade C variant lost (mixture)	Mixture lost variants	Lost variants
> 0	Grade A variant found	0	-	0	Minor Grade A variant lost (mixture)	Mixture lost variants	Lost variants
> 0	Grade B variant found	0	-	0	Minor Grade B variant lost (mixture)	Mixture lost variants	Lost variants
> 0	Grade C variant found	0	-	0	Minor Grade C variant lost (mixture)	Mixture lost variants	Lost variants
> 0	Grade A variant found	> 0	Grade A variant found	0	Shared Grade A variant lost (mixture)	Mixture lost variants	Lost variants
> 0	Grade A variant found	> 0	Grade B variant found	0	Shared Grade B variant lost (mixture)	Mixture lost variants	Lost variants
> 0	Grade B variant found	> 0	Grade A variant found	0	Shared Grade B variant lost (mixture)	Mixture lost variants	Lost variants
> 0	Grade B variant found	> 0	Grade B variant found	0	Shared Grade B variant lost (mixture)	Mixture lost variants	Lost variants
> 0	Grade A variant found	> 0	Grade C variant found	0	Shared Grade C variant lost (mixture)	Mixture lost variants	Lost variants
> 0	Grade B variant found	> 0	Grade C variant found	0	Shared Grade C variant lost (mixture)	Mixture lost variants	Lost variants
> 0	Grade C variant found	> 0	Grade A variant found	0	Shared Grade C variant lost (mixture)	Mixture lost variants	Lost variants
> 0	Grade C variant found	> 0	Grade B variant found	0	Shared Grade C variant lost (mixture)	Mixture lost variants	Lost variants
> 0	Grade C variant found	> 0	Grade C variant found	0	Shared Grade C variant lost (mixture)	Mixture lost variants	Lost variants
0	-	0	Grade A variant lost	0	Major Grade A variant lost (Ion Torrent and mixture)	Old lost variants	Lost variants
0	-	0	Grade B variant lost	0	Major Grade B variant lost (Ion Torrent and mixture)	Old lost variants	Lost variants
0	-	0	Grade C variant lost	0	Major Grade C variant lost (Ion Torrent and mixture)	Old lost variants	Lost variants
0	Grade A variant lost	0	-	0	Minor Grade A variant lost (Ion Torrent and mixture)	Old lost variants	Lost variants
0	Grade B variant lost	0	-	0	Minor Grade B variant lost (Ion Torrent and mixture)	Old lost variants	Lost variants
0	Grade C variant lost	0	-	0	Minor Grade C variant lost (Ion Torrent and mixture)	Old lost variants	Lost variants
0	Grade A variant lost	0	Grade A variant lost	0	Shared Grade A variant lost (Ion Torrent and mixture)	Old lost variants	Lost variants
0	Grade A variant lost	0	Grade B variant lost	0	Shared Grade B variant lost (Ion Torrent and mixture)	Old lost variants	Lost variants
0	Grade B variant lost	0	Grade A variant lost	0	Shared Grade B variant lost (Ion Torrent and mixture)	Old lost variants	Lost variants
0	Grade B variant lost	0	Grade B variant lost	0	Shared Grade B variant lost (Ion Torrent and mixture)	Old lost variants	Lost variants
0	Grade A variant lost	0	Grade C variant lost	0	Shared Grade C variant lost (Ion Torrent and mixture)	Old lost variants	Lost variants
0	Grade B variant lost	0	Grade C variant lost	0	Shared Grade C variant lost (Ion Torrent and mixture)	Old lost variants	Lost variants
0	Grade C variant lost	0	Grade A variant lost	0	Shared Grade C variant lost (Ion Torrent and mixture)	Old lost variants	Lost variants
0	Grade C variant lost	0	Grade B variant lost	0	Shared Grade C variant lost (Ion Torrent and mixture)	Old lost variants	Lost variants
0	Grade C variant lost	0	Grade C variant lost	0	Shared Grade C variant lost (Ion Torrent and mixture)	Old lost variants	Lost variants
0	-	> 0	Novel variant	> 0	Major novel variant found (Ion Torrent and mixture)	Found variants	Novel variants
> 0	Novel variant	0	-	> 0	Minor novel variant found (Ion Torrent and mixture)	Found variants	Novel variants

Minor component: primary analysis VL	Minor component: primary analysis variant class	Major component: primary analysis VL	Major component: primary analysis variant class	Mixture: VL	Mixture: micro	Mixture: meso	Mixture: macro
> 0	Grade A variant found	> 0	Novel variant	> 0	Shared novel variant found (Ion Torrent and mixture)	Found variants	Novel variants
> 0	Grade B variant found	> 0	Novel variant	> 0	Shared novel variant found (Ion Torrent and mixture)	Found variants	Novel variants
> 0	Grade C variant found	> 0	Novel variant	> 0	Shared novel variant found (Ion Torrent and mixture)	Found variants	Novel variants
> 0	Novel variant	> 0	Grade A variant found	> 0	Shared novel variant found (Ion Torrent and mixture)	Found variants	Novel variants
> 0	Novel variant	> 0	Grade B variant found	> 0	Shared novel variant found (Ion Torrent and mixture)	Found variants	Novel variants
> 0	Novel variant	> 0	Grade C variant found	> 0	Shared novel variant found (Ion Torrent and mixture)	Found variants	Novel variants
> 0	Novel variant	> 0	Novel variant	> 0	Shared true novel variant found (Ion Torrent and mixture)	Found variants	Novel variants
0	-	0	-	> 0	Novel variant: mixture	Novel variants	Novel variants
0	-	> 0	Novel variant	0	Major novel variant lost (mixture)	Mixture lost variants	Primary novel variants
> 0	Novel variant	0	-	0	Minor novel variant lost (mixture)	Mixture lost variants	Primary novel variants
> 0	Grade A variant found	> 0	Novel variant	0	Shared novel variant lost (mixture)	Mixture lost variants	Primary novel variants
> 0	Grade B variant found	> 0	Novel variant	0	Shared novel variant lost (mixture)	Mixture lost variants	Primary novel variants
> 0	Grade C variant found	> 0	Novel variant	0	Shared novel variant lost (mixture)	Mixture lost variants	Primary novel variants
> 0	Novel variant	> 0	Grade A variant found	0	Shared novel variant lost (mixture)	Mixture lost variants	Primary novel variants
> 0	Novel variant	> 0	Grade B variant found	0	Shared novel variant lost (mixture)	Mixture lost variants	Primary novel variants
> 0	Novel variant	> 0	Grade C variant found	0	Shared novel variant lost (mixture)	Mixture lost variants	Primary novel variants
> 0	Novel variant	> 0	Novel variant	0	Shared true novel variant lost (mixture)	Mixture lost variants	Primary novel variants
0	Grade A variant lost	> 0	Grade A variant found	> 0	Shared variant found: mixed classification	Shared mixed found variants	Mixed status
0	Grade A variant lost	> 0	Grade B variant found	> 0	Shared variant found: mixed classification	Shared mixed found variants	Mixed status
0	Grade A variant lost	> 0	Grade C variant found	> 0	Shared variant found: mixed classification	Shared mixed found variants	Mixed status
0	Grade A variant lost	> 0	Novel variant	> 0	Shared variant found: mixed classification	Shared mixed found variants	Mixed status
0	Grade B variant lost	> 0	Grade A variant found	> 0	Shared variant found: mixed classification	Shared mixed found variants	Mixed status
0	Grade B variant lost	> 0	Grade B variant found	> 0	Shared variant found: mixed classification	Shared mixed found variants	Mixed status
0	Grade B variant lost	> 0	Grade C variant found	> 0	Shared variant found: mixed classification	Shared mixed found variants	Mixed status
0	Grade B variant lost	> 0	Novel variant	> 0	Shared variant found: mixed classification	Shared mixed found variants	Mixed status
0	Grade C variant lost	> 0	Grade A variant found	> 0	Shared variant found: mixed classification	Shared mixed found variants	Mixed status
0	Grade C variant lost	> 0	Grade B variant found	> 0	Shared variant found: mixed classification	Shared mixed found variants	Mixed status
0	Grade C variant lost	> 0	Grade C variant found	> 0	Shared variant found: mixed classification	Shared mixed found variants	Mixed status
0	Grade C variant lost	> 0	Novel variant	> 0	Shared variant found: mixed classification	Shared mixed found variants	Mixed status
> 0	Grade A variant found	0	Grade A variant lost	> 0	Shared variant found: mixed classification	Shared mixed found variants	Mixed status
> 0	Grade A variant found	0	Grade B variant lost	> 0	Shared variant found: mixed classification	Shared mixed found variants	Mixed status
> 0	Grade A variant found	0	Grade C variant lost	> 0	Shared variant found: mixed classification	Shared mixed found variants	Mixed status

Minor component: primary analysis VL	Minor component: primary analysis variant class	Major component: primary analysis VL	Major component: primary analysis variant class	Mixture: VL	Mixture: micro	Mixture: meso	Mixture: macro
> 0	Grade B variant found	0	Grade A variant lost	> 0	Shared variant found: mixed classification	Shared mixed found variants	Mixed status
> 0	Grade B variant found	0	Grade B variant lost	> 0	Shared variant found: mixed classification	Shared mixed found variants	Mixed status
> 0	Grade B variant found	0	Grade C variant lost	> 0	Shared variant found: mixed classification	Shared mixed found variants	Mixed status
> 0	Grade C variant found	0	Grade A variant lost	> 0	Shared variant found: mixed classification	Shared mixed found variants	Mixed status
> 0	Grade C variant found	0	Grade B variant lost	> 0	Shared variant found: mixed classification	Shared mixed found variants	Mixed status
> 0	Grade C variant found	0	Grade C variant lost	> 0	Shared variant found: mixed classification	Shared mixed found variants	Mixed status
> 0	Novel variant	0	Grade A variant lost	> 0	Shared variant found: mixed classification	Shared mixed found variants	Mixed status
> 0	Novel variant	0	Grade B variant lost	> 0	Shared variant found: mixed classification	Shared mixed found variants	Mixed status
> 0	Novel variant	0	Grade C variant lost	> 0	Shared variant found: mixed classification	Shared mixed found variants	Mixed status
0	Grade A variant lost	> 0	Grade A variant found	0	Shared variant lost: mixed classification	Shared mixed lost variants	Mixed status
0	Grade A variant lost	> 0	Grade B variant found	0	Shared variant lost: mixed classification	Shared mixed lost variants	Mixed status
0	Grade A variant lost	> 0	Grade C variant found	0	Shared variant lost: mixed classification	Shared mixed lost variants	Mixed status
0	Grade A variant lost	> 0	Novel variant	0	Shared variant lost: mixed classification	Shared mixed lost variants	Mixed status
0	Grade B variant lost	> 0	Grade A variant found	0	Shared variant lost: mixed classification	Shared mixed lost variants	Mixed status
0	Grade B variant lost	> 0	Grade B variant found	0	Shared variant lost: mixed classification	Shared mixed lost variants	Mixed status
0	Grade B variant lost	> 0	Grade C variant found	0	Shared variant lost: mixed classification	Shared mixed lost variants	Mixed status
0	Grade B variant lost	> 0	Novel variant	0	Shared variant lost: mixed classification	Shared mixed lost variants	Mixed status
0	Grade C variant lost	> 0	Grade A variant found	0	Shared variant lost: mixed classification	Shared mixed lost variants	Mixed status
0	Grade C variant lost	> 0	Grade B variant found	0	Shared variant lost: mixed classification	Shared mixed lost variants	Mixed status
0	Grade C variant lost	> 0	Grade C variant found	0	Shared variant lost: mixed classification	Shared mixed lost variants	Mixed status
0	Grade C variant lost	> 0	Novel variant	0	Shared variant lost: mixed classification	Shared mixed lost variants	Mixed status
> 0	Grade A variant found	0	Grade A variant lost	0	Shared variant lost: mixed classification	Shared mixed lost variants	Mixed status
> 0	Grade A variant found	0	Grade B variant lost	0	Shared variant lost: mixed classification	Shared mixed lost variants	Mixed status
> 0	Grade A variant found	0	Grade C variant lost	0	Shared variant lost: mixed classification	Shared mixed lost variants	Mixed status
> 0	Grade B variant found	0	Grade A variant lost	0	Shared variant lost: mixed classification	Shared mixed lost variants	Mixed status
> 0	Grade B variant found	0	Grade B variant lost	0	Shared variant lost: mixed classification	Shared mixed lost variants	Mixed status
> 0	Grade B variant found	0	Grade C variant lost	0	Shared variant lost: mixed classification	Shared mixed lost variants	Mixed status
> 0	Grade C variant found	0	Grade A variant lost	0	Shared variant lost: mixed classification	Shared mixed lost variants	Mixed status
> 0	Grade C variant found	0	Grade B variant lost	0	Shared variant lost: mixed classification	Shared mixed lost variants	Mixed status
> 0	Grade C variant found	0	Grade C variant lost	0	Shared variant lost: mixed classification	Shared mixed lost variants	Mixed status
> 0	Novel variant	0	Grade A variant lost	0	Shared variant lost: mixed classification	Shared mixed lost variants	Mixed status
> 0	Novel variant	0	Grade B variant lost	0	Shared variant lost: mixed classification	Shared mixed lost variants	Mixed status

Minor component: primary analysis VL	Minor component: primary analysis variant class	Major component: primary analysis VL	Major component: primary analysis variant class	Mixture: VL	Mixture: micro	Mixture: meso	Mixture: macro
> 0	Novel variant	0	Grade C variant lost	0	Shared variant lost: mixed classification	Shared mixed lost variants	Mixed status
0	-	0	-	0	Not possible	Not possible	Not possible

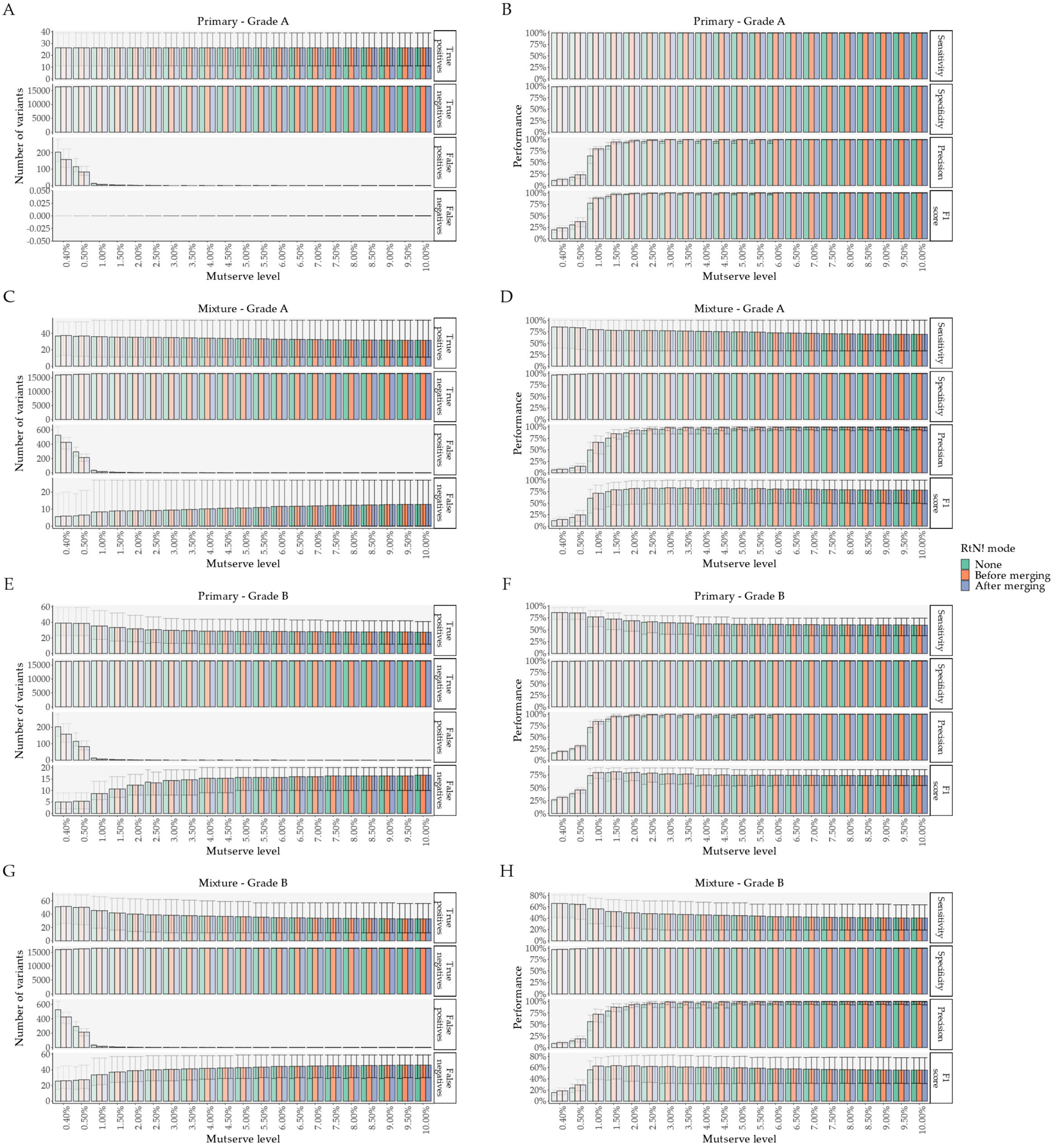

Figure S1 - Optimization of the bioinformatic processing.

(**A, C, E, G**) Performance metric variant classification according to the approach to NUMT removal and heteroplasmy threshold for Primary Grade A, Mixture Grade A, Primary Grade B, and Mixture Grade B datasets, respectively; (**B, D, F, H**) Performance metrics according to the approach to NUMT removal and heteroplasmy threshold for Primary Grade A, Mixture Grade A, Primary Grade B, and Mixture Grade B datasets, respectively. Error bars denote minimum and maximum values.

Table S2 - Optimizing the approach to NUMT removal. Paired t-test with FDR

Performance metric	Grade	RtN! before merged - no RtN!			RtN! before merged - RtN! after merged		
		Estimate [95% CI]	p-value	p-value (adj)	Estimate [95% CI]	p-value	p-value (adj)
F_1 score	Grade A	1.63% [1.32% - 1.93%]	3.14E-22	8.36E-22	0.12% [0.07% - 0.16%]	2.40E-07	1.92E-06
	Grade B	1.03% [0.81% - 1.25%]	5.52E-18	1.10E-17	0.05% [0.03% - 0.07%]	5.36E-05	7.14E-05
Sensitivity	Grade A	-0.23% [-0.33% - -0.13%]	7.33E-06	7.33E-06	0.01% [-0.01% - 0.02%]	3.18E-01	3.64E-01
	Grade B	-0.16% [-0.23% - -0.10%]	1.40E-06	1.60E-06	0.00% [-0.02% - 0.01%]	6.38E-01	6.38E-01
Specificity	Grade A	0.06% [0.04% - 0.08%]	8.33E-11	1.11E-10	0.00% [0.00% - 0.00%]	5.96E-06	1.52E-05
	Grade B	0.06% [0.04% - 0.08%]	8.34E-11	1.11E-10	0.00% [0.00% - 0.00%]	5.95E-06	1.52E-05
Precision	Grade A	3.32% [2.82% - 3.82%]	1.73E-31	6.91E-31	0.50% [0.28% - 0.71%]	9.51E-06	1.52E-05
	Grade B	3.12% [2.66% - 3.58%]	2.94E-32	2.35E-31	0.46% [0.26% - 0.65%]	7.61E-06	1.52E-05

Table S3 - Optimizing the heteroplasmy threshold in mutserve, in datasets processed with RtN! before merging

Mutserve level	Primary - Grade A	Mixture - Grade A	Primary - Grade B	Mixture - Grade B	Mean
2.50%	99.58%	82.94%	78.56%	62.20%	80.82%
2.00%	98.47%	81.76%	79.41%	63.00%	80.66%
3.00%	100.00%	83.49%	77.08%	62.01%	80.65%
3.50%	100.00%	83.28%	76.72%	61.27%	80.32%
1.50%	97.26%	79.10%	81.08%	63.52%	80.24%
4.00%	100.00%	82.93%	75.10%	60.75%	79.70%
4.50%	100.00%	82.50%	75.10%	60.20%	79.45%
5.00%	100.00%	82.41%	74.55%	59.82%	79.20%
5.50%	100.00%	81.94%	74.55%	59.02%	78.88%
6.00%	100.00%	80.90%	74.55%	57.95%	78.35%
6.50%	100.00%	80.69%	74.18%	57.76%	78.16%
7.00%	100.00%	80.23%	74.18%	57.14%	77.89%
7.50%	100.00%	79.63%	73.80%	56.69%	77.53%
8.00%	100.00%	79.25%	73.80%	56.35%	77.35%
8.50%	100.00%	78.97%	73.80%	56.08%	77.21%
9.00%	100.00%	78.63%	73.80%	55.82%	77.06%
9.50%	100.00%	78.51%	73.80%	55.58%	76.97%
10.00%	100.00%	78.51%	73.41%	55.58%	76.87%
1.00%	88.78%	71.70%	79.61%	62.83%	75.73%
0.50%	37.41%	24.51%	45.78%	29.20%	34.23%
0.40%	23.85%	14.53%	31.54%	18.47%	22.10%

Table S4 - Primary analysis: Overall look

Sample	Sequencing run		Mean coverage	Mappability >30 reads/base	Mappability >100 reads/base	Mappability >1000 reads/base	Number of variants	Haplogroup	Haplogroup quality (%)
HG00256 (*H*)	exome		29.10	37.84%	0.00%	0.00%	12	H5b2	98.98%
	lowCov		4172.55	100.00%	100.00%	99.95%	42	H5b2	98.98%
	highCov		16370.49	100.00%	100.00%	99.99%	85	H5b2	95.80%
	Ion Torrent	PCP	932.28	99.29%	96.80%	42.07%	14	H5b2	98.98%
		TSS	1342.00	-	-	-	12	H5b2	98.98%
HG01626 (*T*)	exome		72.86	71.85%	11.86%	0.86%	42	T2a1b1a1b	97.07%
	lowCov		2641.51	100.00%	99.99%	98.91%	61	T2a1b1a1b	97.60%
	highCov		14422.54	100.00%	100.00%	99.99%	127	T2a1b1a1b	97.60%
	Ion Torrent	PCP	3196.55	99.98%	99.91%	87.91%	48	T2a1b1a1b	97.60%
		TSS	4256.00	-	-	-	40	T2a1b1a1b	94.94%
HG01757 (*U*)	exome		1159.80	99.99%	99.99%	58.89%	40	U4a	95.92%
	lowCov		4776.03	100.00%	100.00%	99.99%	41	U4a	95.92%
	highCov		14444.80	100.00%	100.00%	99.99%	95	U4a	94.33%
	Ion Torrent	PCP	1115.99	99.90%	95.59%	49.45%	31	U4a	95.92%
		TSS	1577.00	-	-	-	31	U4a	95.92%

Table S5 - Ion Torrent sequencing: Overall look on differences between PCP and TSS

Sample	PCP							TSS						
	Haplogroup	Haplogroup quality (%)	Contamination	Contamination level	Number of sequences	Mean coverage	Number of variants	Haplogroup	Haplogroup quality (%)	Contamination	Contamination level	Number of sequences	Mean coverage	Number of variants
H	H5b2	98.98%	No	-	144178	932.28	14	H5b2	98.98%	No	-	184905	1342.00	12
U0.01H			No	-	177989	1163.89	14			No	-	214373	1567.00	13
U0.02H			No	-	245198	1596.93	14			No	-	292422	2131.00	13
U0.05H			No	-	245786	1636.51	14			No	-	297105	2206.00	13
U0.10H		96.53%	Yes	7.50%	248124	1612.49	35		96.53%	No	-	291568	2122.00	14
U0.25H	H5'36	98.57%	Yes	31.30%	265832	1730.69	33	H+195	100.00%	Yes	29.00%	318735	2325.00	36
T	T2a1b1a1b	97.60%	No	-	494101	3196.55	48	T2a1b1a1b	94.94%	Yes	0.80%	586187	4256.00	40
H0.01T			No	-	168591	1098.88	46			No	-	205371	1494.00	41
H0.02T			No	-	177652	1144.59	48			No	-	217075	1569.00	42
H0.05T			No	-	429697	2802.03	45			No	-	502798	3672.00	41
H0.10T			Yes	6.30%	329892	2158.23	53			No	-	384980	2825.00	43
H0.25T	H	100.00%	Yes	18.80%	360527	2347.62	51	H	100.00%	Yes	18.90%	428989	3141.00	47
U	U4a	95.92%	No	-	172011	1115.99	31	U4a	95.92%	Yes	0.60%	216643	1577.00	31
T0.01U			No	-	191343	1250.31	31			No	-	231015	1687.00	32
T0.02U		94.69%	No	-	163090	1062.47	31			No	-	196335	1432.00	30
T0.05U		93.99%	Yes	4.40%	211826	1373.27	59			No	-	253305	1835.00	31
T0.10U	R	94.91%	Yes	13.40%	255253	1656.45	60	R	100.00%	Yes	13.40%	308232	2240.00	50
T0.25U	R	98.75%	Yes	36.60%	258009	1676.07	62	R		Yes	36.60%	307354	2242.00	55

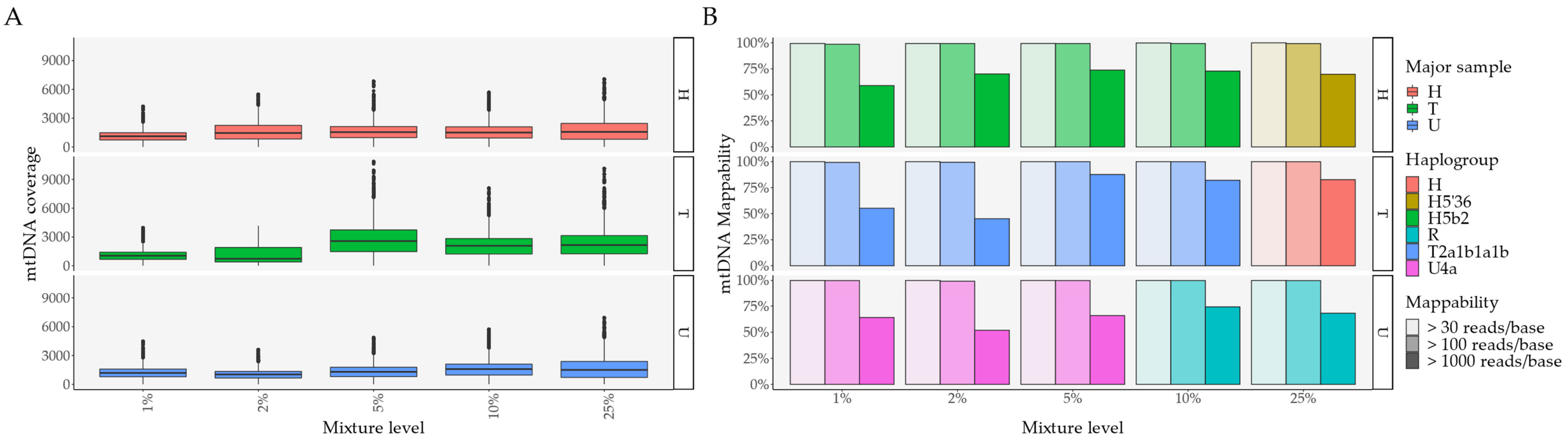

Figure S2 - Mixture analysis: Overall look with PCP, in mutserve.

(**A**) Coverage per sample, (**B**) Mappability and haplogroup assignment per sample.

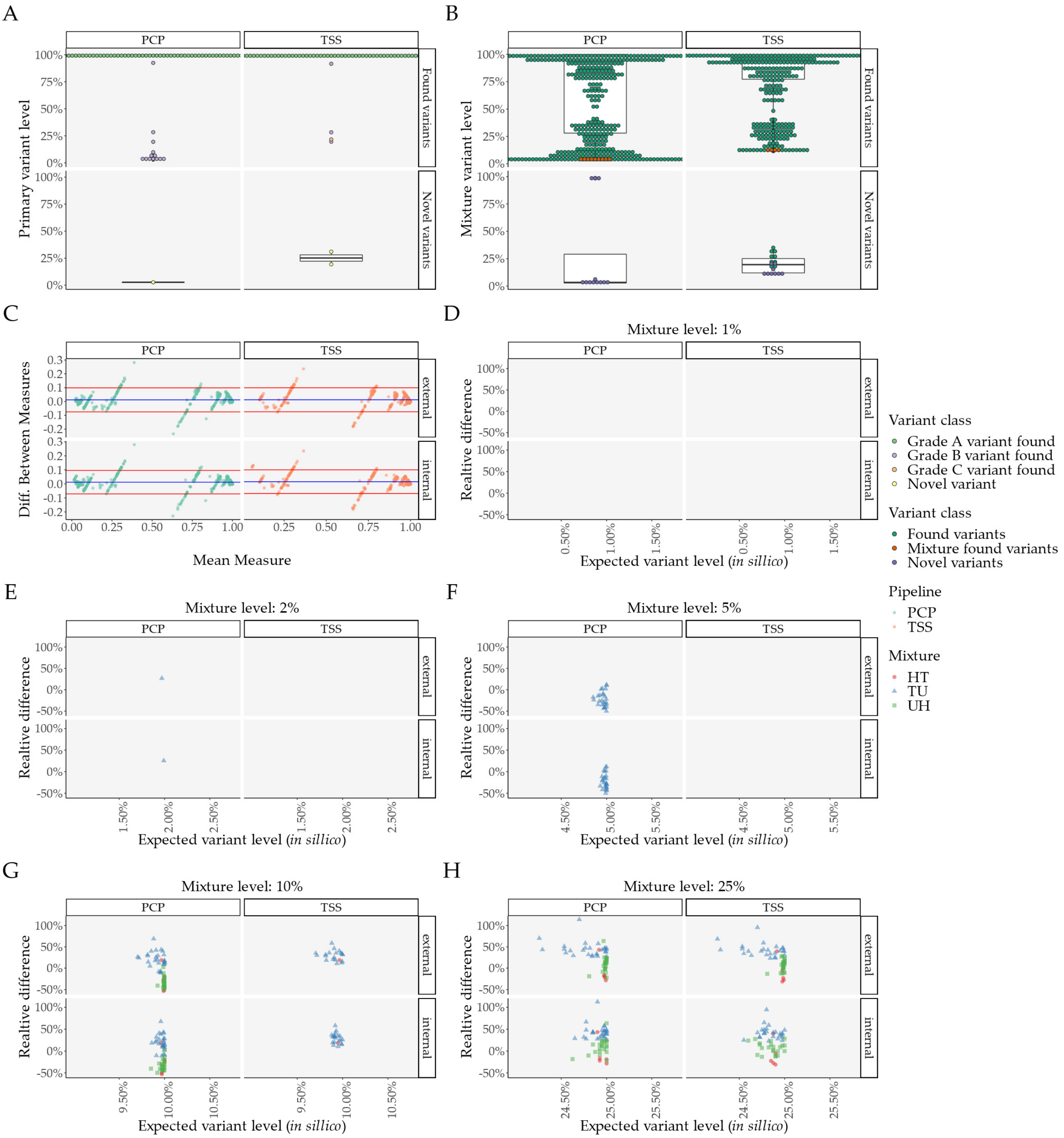

Figure S3 - Distribution of variant levels with mutserve, per pipeline.

(**A**) Primary analysis - distribution of variant levels per variant classification, per pipeline, (**B**) Mixture analysis - distribution of variant levels per macro variant classification, per pipeline, (**C**) Bland–Altman plot for internal and external differences, per pipeline; (**D-H**) close-up of Figure 4A with the relative difference between expected and observed variant level for each internal and external comparison, per pipeline and per mixture, depending on mixture level: 1%, 2%, 5%, 10%, and 25%, respectively.

Table S6 - Primary analysis: Distribution of Grade A/B variants, according to the VCM chosen. McNemar's test with FDR

VCM	Variants found with PCP and TSS	Variants found with PCP & lost with TSS	Variants lost with PCP & found with TSS	Variants lost with PCP and TSS	p-value	p-value (adj)
freebayes	70	9	1	44	2.69E-02	4.03E-02
mutserve	80	12	0	40	1.50E-03	4.49E-03
varscan	75	11	5	33	2.11E-01	2.11E-01

Table S7 - Primary analysis: Paired difference between the observed VL in Ion Torrent and the expected VL in Grade A/B variants with other sequencing methods, according to the VCM chosen. Bootstrap with FDR

VCM	Mean TSS-PCP paired difference [95% CI]	p-value	p-value (adj)
freebayes	0.33% [0.12% - 0.50%]	0.00E+00	0.00E+00
mutserve	0.30% [0.13% - 0.41%]	0.00E+00	0.00E+00
varscan	0.32% [0.14% - 0.40%]	0.00E+00	0.00E+00

Table S8 - Primary analysis: Paired difference between the proportion of "Novel variants", depending on the VCM chosen. Bootstrap with FDR

VCM	Mean TSS-PCP paired difference [95% CI]	p-value	p-value (adj)
freebayes	1.05% [0.00% - 2.44%]	7.47E-02	2.24E-01
mutserve	0.38% [-0.78% - 1.92%]	5.29E-01	5.29E-01
varscan	1.04% [0.00% - 3.12%]	5.05E-01	5.29E-01

Table S9 - Mixture analysis: Distribution of Grade A/B variants, according to the VCM chosen. McNemar's test with FDR

VCM	Variants found with PCP and TSS	Variants found with PCP & lost with TSS	Variants lost with PCP & found with TSS	Variants lost with PCP and TSS	p-value	p-value (adj)
freebayes	409	80	11	570	1.02E-12	1.02E-12
mutserve	477	107	1	600	5.32E-24	1.60E-23
varscan	457	102	21	535	5.46E-13	8.19E-13

Table S10 - Mixture analysis: Paired difference between the observed VL and the expected VL (internal and external) in Grade A/B variants, according to the VCM chosen. Paired t-test with FDR

Macro variant description	VCM	Comparison	Calculation	Mean paired difference [95% CI]	p-value	p-value (adj)
Found variants	freebayes	internal (within plaftorm)	mean(PCP - expected VL)	1.20% [0.39% - 2.02%]	6.89E-03	1.13E-02
			mean(TSS - expected VL)	1.55% [0.87% - 2.22%]	2.27E-04	8.16E-04
			mean[mean(TSS - expected VL) - mean(PCP - expected VL)]	0.35% [-0.15% - 0.84%]	1.55E-01	1.99E-01
		external (other plaftorms)	mean(PCP - expected VL)	1.07% [0.22% - 1.93%]	1.77E-02	2.65E-02
			mean(TSS - expected VL)	1.14% [0.38% - 1.90%]	6.08E-03	1.11E-02
			mean[mean(TSS - expected VL) - mean(PCP - expected VL)]	0.07% [-0.37% - 0.51%]	7.42E-01	7.86E-01
	mutserve	internal (within plaftorm)	mean(PCP - expected VL)	1.15% [0.38% - 1.92%]	6.56E-03	1.12E-02
			mean(TSS - expected VL)	1.46% [0.82% - 2.10%]	2.26E-04	8.16E-04
			mean[mean(TSS - expected VL) - mean(PCP - expected VL)]	0.31% [-0.17% - 0.79%]	1.88E-01	2.33E-01
		external (other plaftorms)	mean(PCP - expected VL)	1.02% [0.21% - 1.82%]	1.68E-02	2.63E-02
			mean(TSS - expected VL)	1.07% [0.37% - 1.77%]	5.35E-03	1.11E-02
			mean[mean(TSS - expected VL) - mean(PCP - expected VL)]	0.06% [-0.38% - 0.50%]	7.75E-01	7.97E-01
	varscan	internal (within plaftorm)	mean(PCP - expected VL)	2.46% [1.42% - 3.50%]	1.75E-04	8.16E-04

Macro variant description	VCM	Comparison	Calculation	Mean paired difference [95% CI]	p-value	p-value (adj)
			mean(TSS - expected VL)	1.46% [0.82% - 2.10%]	2.26E-04	8.16E-04
			mean[mean(TSS - expected VL) - mean(PCP - expected VL)]	-1.00% [-1.93% - -0.06%]	3.81E-02	5.08E-02
		external (other plaftorms)	mean(PCP - expected VL)	1.03% [0.20% - 1.87%]	1.90E-02	2.66E-02
			mean(TSS - expected VL)	1.05% [0.35% - 1.76%]	6.19E-03	1.11E-02
			mean[mean(TSS - expected VL) - mean(PCP - expected VL)]	0.02% [-0.46% - 0.50%]	9.31E-01	9.31E-01
Lost variants	freebayes	internal (within plaftorm)	mean(PCP - expected VL)	-1.45% [-2.14% - -0.75%]	5.27E-04	1.46E-03
			mean(TSS - expected VL)	-0.92% [-1.52% - -0.31%]	5.84E-03	1.11E-02
			mean[mean(TSS - expected VL) - mean(PCP - expected VL)]	0.53% [-0.34% - 1.40%]	2.12E-01	2.46E-01
		external (other plaftorms)	mean(PCP - expected VL)	-2.59% [-3.11% - -2.06%]	4.71E-08	1.70E-06
			mean(TSS - expected VL)	-1.55% [-2.11% - -0.99%]	3.77E-05	2.26E-04
			mean[mean(TSS - expected VL) - mean(PCP - expected VL)]	1.04% [0.20% - 1.88%]	1.92E-02	2.66E-02
	mutserve	internal (within plaftorm)	mean(PCP - expected VL)	-0.66% [-0.96% - -0.36%]	3.68E-04	1.20E-03
			mean(TSS - expected VL)	-1.00% [-1.63% - -0.37%]	4.31E-03	9.69E-03
			mean[mean(TSS - expected VL) - mean(PCP - expected VL)]	-0.34% [-1.00% - 0.31%]	2.84E-01	3.19E-01
		external (other plaftorms)	mean(PCP - expected VL)	-0.92% [-1.24% - -0.61%]	2.22E-05	1.75E-04
			mean(TSS - expected VL)	-3.08% [-4.16% - -2.01%]	2.44E-05	1.75E-04
			mean[mean(TSS - expected VL) - mean(PCP - expected VL)]	-2.16% [-3.36% - -0.96%]	1.71E-03	4.40E-03
	varscan	internal (within plaftorm)	mean(PCP - expected VL)	-0.65% [-0.96% - -0.34%]	5.01E-04	1.46E-03

Macro variant description	VCM	Comparison	Calculation	Mean paired difference [95% CI]	p-value	p-value (adj)
			mean(TSS - expected VL)	-1.11% [-1.81% - -0.41%]	4.29E-03	9.69E-03
			mean[mean(TSS - expected VL) - mean(PCP - expected VL)]	-0.46% [-1.21% - 0.28%]	2.06E-01	2.46E-01
		external (other plaftorms)	mean(PCP - expected VL)	-4.10% [-5.38% - -2.81%]	8.09E-06	1.46E-04
			mean(TSS - expected VL)	-3.39% [-4.56% - -2.21%]	2.35E-05	1.75E-04
			mean[mean(TSS - expected VL) - mean(PCP - expected VL)]	0.71% [-1.38% - 2.79%]	4.79E-01	5.22E-01

Table S11 - Mixture analysis: Paired relative difference between the observed VL and the expected VL (internal and external) in Grade A/B variants, according to the VCM chosen. Paired t-test with FDR

Macro variant description	VCM	Comparison	Calculation	Mean paired difference [95% CI]	p-value	p-value (adj)
Found variants	freebayes	internal (within plaftorm)	mean[(PCP - expected VL)/expected VL]	1.82% [-3.26% - 6.90%]	4.55E-01	5.08E-01
			mean[(TSS - expected VL)/expected VL]	3.47% [0.94% - 6.00%]	1.07E-02	6.40E-02
			mean{mean[(PCP - expected VL)/expected VL] - mean[(TSS - expected VL)/expected VL]}	1.65% [-2.05% - 5.36%]	3.54E-01	5.08E-01
		external (other plaftorms)	mean[(PCP - expected VL)/expected VL]	1.81% [-3.19% - 6.81%]	4.51E-01	5.08E-01
			mean[(TSS - expected VL)/expected VL]	2.95% [0.29% - 5.61%]	3.24E-02	9.71E-02
			mean{mean[(PCP - expected VL)/expected VL] - mean[(TSS - expected VL)/expected VL]}	1.14% [-2.40% - 4.68%]	5.01E-01	5.08E-01
	mutserve	internal (within plaftorm)	mean[(PCP - expected VL)/expected VL]	1.62% [-3.19% - 6.43%]	4.82E-01	5.08E-01
			mean[(TSS - expected VL)/expected VL]	3.29% [1.01% - 5.57%]	7.85E-03	6.40E-02
			mean{mean[(PCP - expected VL)/expected VL] - mean[(TSS - expected VL)/expected VL]}	1.67% [-1.97% - 5.31%]	3.41E-01	5.08E-01
		external (other plaftorms)	mean[(PCP - expected VL)/expected VL]	1.50% [-3.23% - 6.23%]	5.07E-01	5.08E-01
			mean[(TSS - expected VL)/expected VL]	2.78% [0.40% - 5.16%]	2.52E-02	9.48E-02
			mean{mean[(PCP - expected VL)/expected VL] - mean[(TSS - expected VL)/expected VL]}	1.28% [-2.21% - 4.77%]	4.46E-01	5.08E-01

Macro variant description	VCM	Comparison	Calculation	Mean paired difference [95% CI]	p-value	p-value (adj)
	varscan	internal (within plaftorm)	mean[(PCP - expected VL)/expected VL]	1.74% [-3.33% - 6.80%]	4.74E-01	5.08E-01
			mean[(TSS - expected VL)/expected VL]	3.29% [1.01% - 5.57%]	7.85E-03	6.40E-02
			mean{mean[(PCP - expected VL)/expected VL] - mean[(TSS - expected VL)/expected VL]}	1.55% [-2.31% - 5.41%]	4.03E-01	5.08E-01
		external (other plaftorms)	mean[(PCP - expected VL)/expected VL]	1.61% [-3.29% - 6.50%]	4.93E-01	5.08E-01
			mean[(TSS - expected VL)/expected VL]	2.75% [0.37% - 5.13%]	2.63E-02	9.48E-02
			mean{mean[(PCP - expected VL)/expected VL] - mean[(TSS - expected VL)/expected VL]}	1.15% [-2.48% - 4.77%]	5.08E-01	5.08E-01

Table S12 - Mixture analysis: Paired difference between correlation indicators (internal and external) in Grade A/B variants, depending on the VCM chosen. Paired t-test with FDR

VCM	Variant grade	Comparison	Mean paired difference [95% CI]	p-value	p-value (adj)
freebayes	A	PCP internal - PCP external	0.59 [0.07 - 1.11]	2.79E-02	7.44E-02
		TSS internal - TSS external	0.28 [-0.05 - 0.61]	9.31E-02	1.60E-01
		TSS internal - PCP internal	-5.77 [-13.12 - 1.58]	1.15E-01	1.72E-01
		TSS external - PCP external	-5.45 [-12.63 - 1.73]	1.26E-01	1.74E-01
	B	PCP internal - PCP external	0.77 [-0.26 - 1.79]	1.30E-01	1.74E-01
		TSS internal - TSS external	0.11 [-1.16 - 1.39]	8.50E-01	8.50E-01
		TSS internal - PCP internal	-16.23 [-25.82 - -6.64]	2.73E-03	9.37E-03
		TSS external - PCP external	-15.57 [-25.24 - -5.91]	3.86E-03	1.16E-02
mutserve	A	PCP internal - PCP external	0.34 [-0.04 - 0.72]	7.70E-02	1.51E-01
		TSS internal - TSS external	1.37 [0.69 - 2.06]	7.48E-04	7.84E-03
		TSS internal - PCP internal	-10.65 [-22.82 - 1.52]	8.16E-02	1.51E-01
		TSS external - PCP external	-11.68 [-23.90 - 0.53]	5.94E-02	1.30E-01
	B	PCP internal - PCP external	0.38 [-0.64 - 1.40]	4.35E-01	5.22E-01
		TSS internal - TSS external	0.25 [-1.02 - 1.52]	6.81E-01	7.78E-01

VCM	Variant grade	Comparison	Mean paired difference [95% CI]	p-value	p-value (adj)
		TSS internal - PCP internal	-20.98 [-32.15 - -9.81]	1.25E-03	7.84E-03
		TSS external - PCP external	-20.84 [-32.31 - -9.38]	1.61E-03	7.84E-03
varscan	A	PCP internal - PCP external	-10.82 [-20.92 - -0.72]	3.76E-02	9.02E-02
		TSS internal - TSS external	1.29 [0.60 - 1.98]	1.32E-03	7.84E-03
		TSS internal - PCP internal	3.22 [-4.92 - 11.35]	4.11E-01	5.19E-01
		TSS external - PCP external	-8.89 [-20.02 - 2.23]	1.09E-01	1.72E-01
	B	PCP internal - PCP external	-20.89 [-32.54 - -9.23]	1.79E-03	7.84E-03
		TSS internal - TSS external	0.16 [-1.08 - 1.40]	7.85E-01	8.19E-01
		TSS internal - PCP internal	1.66 [-8.28 - 11.60]	7.25E-01	7.91E-01
		TSS external - PCP external	-19.38 [-30.33 - -8.44]	1.96E-03	7.84E-03

Table S13 - Mixture analysis: Paired difference between the proportion of “Novel variants”, depending on the VCM chosen. Paired t-test with FDR

VCM	Mean TSS-PCP paired difference [95% CI]	p-value	p-value (adj)
freebayes	-0.52% [-0.81% - -0.24%]	1.58E-03	4.74E-03
mutserve	-0.12% [-0.41% - 0.17%]	4.02E-01	6.03E-01
varscan	-0.07% [-0.47% - 0.32%]	6.94E-01	6.94E-01

Table S14 - Mixture analysis: Paired difference between TSS and PCP VL for “Novel variants”, depending on the VCM chosen. Paired t-test with FDR

VCM	Comparison	Mean TSS-PCP paired difference [95% CI]	p-value	p-value (adj)
freebayes	Found novel	68.58% [59.44% - 77.73%]	2.02E-10	1.21E-09
	New novel	3.85% [-0.68% - 8.38%]	8.89E-02	1.07E-01
mutserve	Found novel	26.69% [21.65% - 31.73%]	4.78E-06	9.57E-06
	New novel	-7.03% [-29.49% - 15.43%]	5.01E-01	5.01E-01
varscan	Found novel	27.38% [24.09% - 30.66%]	4.31E-10	1.29E-09
	New novel	7.51% [2.36% - 12.67%]	8.77E-03	1.31E-02

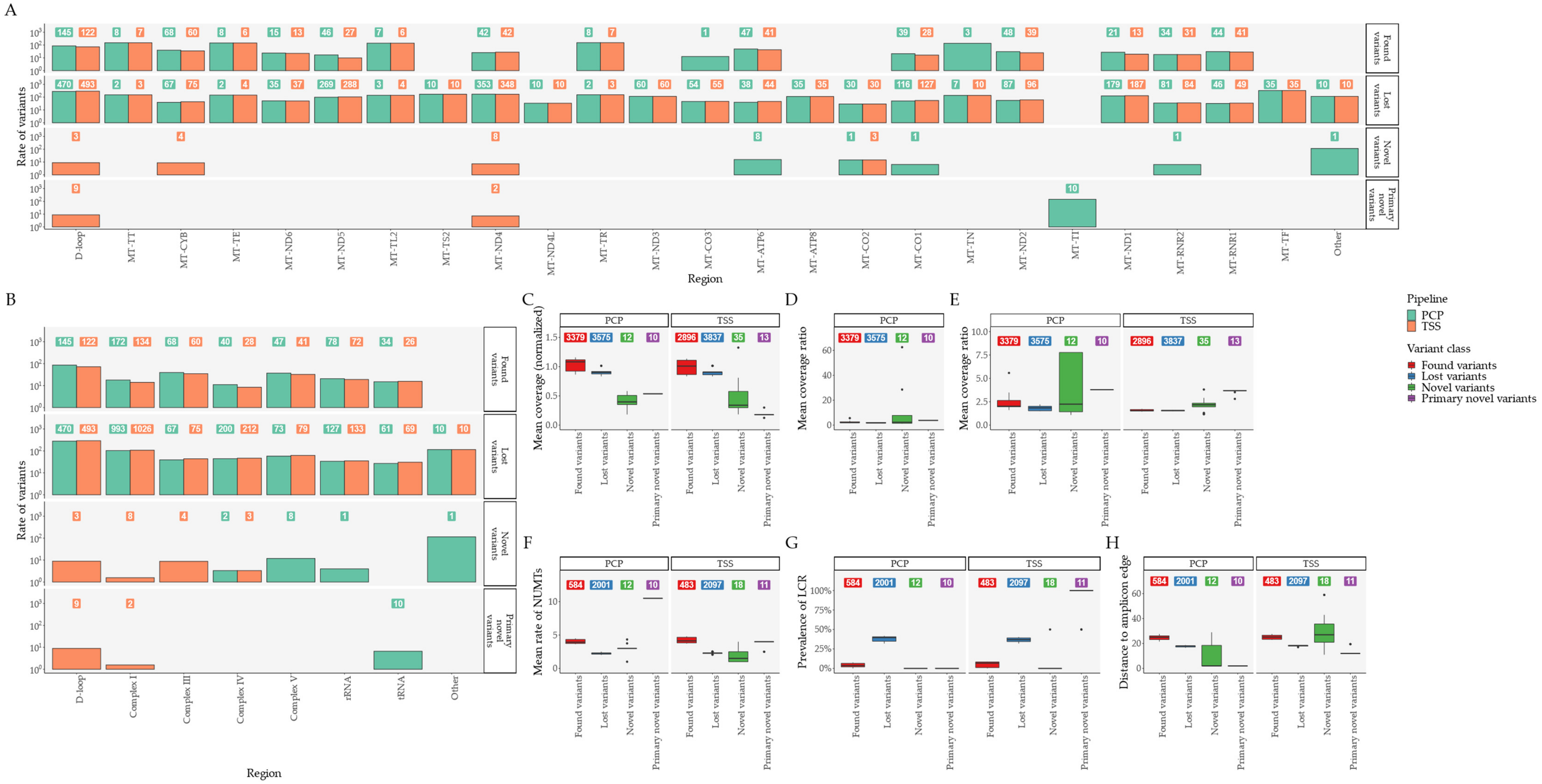

Figure S4 - Differences between different variant classifications, per pipeline.

(**A**) Relative variant burden of different mtDNA regions, per pipeline and per variant classification; (**B**) Relative variant burden of different macro mtDNA regions, per pipeline and per variant classification; (**C**) Mean normalized coverage per sample, per pipeline and per variant classification; (**D**) Mean coverage ratio per sample in PCP, per variant classification; (**E**) Mean coverage ratio per sample, per pipeline and per variant classification, with a close-up of Figure S4D regarding PCP; (**F**) Mean rate of NUMTs per sample, per pipeline and per variant classification; (**G**) Mean prevalence of low complexity region (LCR) variants per sample, per pipeline and per variant classification; (**H**) Mean distance to "callable" amplicon edge per sample, per pipeline and per variant classification. Labels above the columns/boxplots represent the number of variants for each x-value. Region *Other* refers to positions in rCRS with an overlap between the two strands or unannotated. For the mean normalized coverage and for the mean coverage ratio, we considered all variants from both the primary and the mixture analysis, including the output from the Illumina sequencing; otherwise, it would not be possible to have coverage and its ratio for false negatives.

Table S15 - Mixture analysis: Relative burden of different macro variants in different mtDNA regions, depending on the VCM chosen. All regions

VCM	Pipeline	Macro variant description	Macro region	Region	Relative burden
freebayes	PCP	Found variants	Complex I	MT-ND1	27.46
freebayes	PCP	Found variants	Complex I	MT-ND2	30.71
freebayes	PCP	Found variants	Complex I	MT-ND4	28.13
freebayes	PCP	Found variants	Complex I	MT-ND5	11.04
freebayes	PCP	Found variants	Complex I	MT-ND6	23.81
freebayes	PCP	Found variants	Complex III	MT-CYB	39.73
freebayes	PCP	Found variants	Complex IV	MT-CO1	21.08
freebayes	PCP	Found variants	Complex IV	MT-CO3	12.77
freebayes	PCP	Found variants	Complex V	MT-ATP6	33.60
freebayes	PCP	Found variants	D-loop	D-loop	66.55
freebayes	PCP	Found variants	rRNA	MT-RNR1	30.75
freebayes	PCP	Found variants	rRNA	MT-RNR2	17.66
freebayes	PCP	Found variants	tRNA	MT-TE	144.93
freebayes	PCP	Found variants	tRNA	MT-TL2	140.85
freebayes	PCP	Found variants	tRNA	MT-TN	136.99
freebayes	PCP	Found variants	tRNA	MT-TR	153.85
freebayes	PCP	Found variants	tRNA	MT-TT	151.52
freebayes	PCP	Lost variants	Complex I	MT-ND1	205.02
freebayes	PCP	Lost variants	Complex I	MT-ND2	46.07
freebayes	PCP	Lost variants	Complex I	MT-ND3	144.51
freebayes	PCP	Lost variants	Complex I	MT-ND4	199.85
freebayes	PCP	Lost variants	Complex I	MT-ND4L	33.67
freebayes	PCP	Lost variants	Complex I	MT-ND5	134.29
freebayes	PCP	Lost variants	Complex I	MT-ND6	36.63
freebayes	PCP	Lost variants	Complex III	MT-CYB	53.75
freebayes	PCP	Lost variants	Complex IV	MT-CO1	41.50
freebayes	PCP	Lost variants	Complex IV	MT-CO2	19.49
freebayes	PCP	Lost variants	Complex IV	MT-CO3	58.75
freebayes	PCP	Lost variants	Complex V	MT-ATP6	66.14
freebayes	PCP	Lost variants	Complex V	MT-ATP8	177.13
freebayes	PCP	Lost variants	D-loop	D-loop	251.34
freebayes	PCP	Lost variants	rRNA	MT-RNR1	39.13
freebayes	PCP	Lost variants	rRNA	MT-RNR2	41.53
freebayes	PCP	Lost variants	tRNA	MT-TE	144.93
freebayes	PCP	Lost variants	tRNA	MT-TF	422.54
freebayes	PCP	Lost variants	tRNA	MT-TL1	133.33
freebayes	PCP	Lost variants	tRNA	MT-TL2	140.85
freebayes	PCP	Lost variants	tRNA	MT-TN	136.99
freebayes	PCP	Lost variants	tRNA	MT-TR	153.85
freebayes	PCP	Lost variants	tRNA	MT-TT	151.52
freebayes	PCP	Lost variants	tRNA	MT-TW	147.06
freebayes	PCP	Novel variants	Complex I	MT-ND4	7.29
freebayes	PCP	Novel variants	Complex I	MT-ND5	5.52
freebayes	PCP	Novel variants	Complex IV	MT-CO2	14.62
freebayes	PCP	Novel variants	Complex V	MT-ATP6	17.06
freebayes	PCP	Novel variants	tRNA	MT-TE	144.93
freebayes	PCP	Primary novel variants	Complex V	MT-ATP6	15.75
freebayes	PCP	Primary novel variants	tRNA	MT-TE	144.93

VCM	Pipeline	Macro variant description	Macro region	Region	Relative burden
freebayes	PCP	Primary novel variants	tRNA	MT-TI	144.93
freebayes	TSS	Found variants	Complex I	MT-ND1	19.43
freebayes	TSS	Found variants	Complex I	MT-ND2	24.95
freebayes	TSS	Found variants	Complex I	MT-ND4	24.53
freebayes	TSS	Found variants	Complex I	MT-ND5	9.93
freebayes	TSS	Found variants	Complex I	MT-ND6	22.51
freebayes	TSS	Found variants	Complex III	MT-CYB	35.06
freebayes	TSS	Found variants	Complex IV	MT-CO1	16.51
freebayes	TSS	Found variants	Complex V	MT-ATP6	35.79
freebayes	TSS	Found variants	D-loop	D-loop	65.36
freebayes	TSS	Found variants	rRNA	MT-RNR1	28.65
freebayes	TSS	Found variants	rRNA	MT-RNR2	18.10
freebayes	TSS	Found variants	tRNA	MT-TE	144.93
freebayes	TSS	Found variants	tRNA	MT-TL2	140.85
freebayes	TSS	Found variants	tRNA	MT-TR	153.85
freebayes	TSS	Found variants	tRNA	MT-TT	151.52
freebayes	TSS	Lost variants	Complex I	MT-ND1	210.60
freebayes	TSS	Lost variants	Complex I	MT-ND2	51.82
freebayes	TSS	Lost variants	Complex I	MT-ND3	144.51
freebayes	TSS	Lost variants	Complex I	MT-ND4	208.12
freebayes	TSS	Lost variants	Complex I	MT-ND4L	33.67
freebayes	TSS	Lost variants	Complex I	MT-ND5	135.39
freebayes	TSS	Lost variants	Complex I	MT-ND6	36.73
freebayes	TSS	Lost variants	Complex III	MT-CYB	58.43
freebayes	TSS	Lost variants	Complex IV	MT-CO1	46.26
freebayes	TSS	Lost variants	Complex IV	MT-CO2	19.49
freebayes	TSS	Lost variants	Complex IV	MT-CO3	59.60
freebayes	TSS	Lost variants	Complex V	MT-ATP6	62.99
freebayes	TSS	Lost variants	Complex V	MT-ATP8	177.13
freebayes	TSS	Lost variants	D-loop	D-loop	252.53
freebayes	TSS	Lost variants	rRNA	MT-RNR1	41.23
freebayes	TSS	Lost variants	rRNA	MT-RNR2	42.39
freebayes	TSS	Lost variants	tRNA	MT-TE	144.93
freebayes	TSS	Lost variants	tRNA	MT-TF	422.54
freebayes	TSS	Lost variants	tRNA	MT-TL1	133.33
freebayes	TSS	Lost variants	tRNA	MT-TL2	140.85
freebayes	TSS	Lost variants	tRNA	MT-TN	136.99
freebayes	TSS	Lost variants	tRNA	MT-TR	153.85
freebayes	TSS	Lost variants	tRNA	MT-TT	151.52
freebayes	TSS	Lost variants	tRNA	MT-TW	147.06
freebayes	TSS	Novel variants	Complex I	MT-ND4	7.29
freebayes	TSS	Novel variants	Complex III	MT-CYB	8.76
freebayes	TSS	Novel variants	Complex IV	MT-CO2	14.62
freebayes	TSS	Novel variants	Complex V	MT-ATP6	25.20
freebayes	TSS	Novel variants	D-loop	D-loop	19.10
freebayes	TSS	Primary novel variants	Complex I	MT-ND4	7.29
freebayes	TSS	Primary novel variants	Complex V	MT-ATP6	15.75
freebayes	TSS	Primary novel variants	D-loop	D-loop	16.84
mutserve	PCP	Found variants	Complex I	MT-ND1	27.46

VCM	Pipeline	Macro variant description	Macro region	Region	Relative burden
mutserve	PCP	Found variants	Complex I	MT-ND2	30.71
mutserve	PCP	Found variants	Complex I	MT-ND4	25.53
mutserve	PCP	Found variants	Complex I	MT-ND5	16.92
mutserve	PCP	Found variants	Complex I	MT-ND6	23.81
mutserve	PCP	Found variants	Complex III	MT-CYB	39.73
mutserve	PCP	Found variants	Complex IV	MT-CO1	21.08
mutserve	PCP	Found variants	Complex IV	MT-CO3	12.77
mutserve	PCP	Found variants	Complex V	MT-ATP6	49.34
mutserve	PCP	Found variants	D-loop	D-loop	86.16
mutserve	PCP	Found variants	rRNA	MT-RNR1	30.75
mutserve	PCP	Found variants	rRNA	MT-RNR2	18.20
mutserve	PCP	Found variants	tRNA	MT-TE	144.93
mutserve	PCP	Found variants	tRNA	MT-TL2	140.85
mutserve	PCP	Found variants	tRNA	MT-TN	136.99
mutserve	PCP	Found variants	tRNA	MT-TR	153.85
mutserve	PCP	Found variants	tRNA	MT-TT	151.52
mutserve	PCP	Lost variants	Complex I	MT-ND1	124.83
mutserve	PCP	Lost variants	Complex I	MT-ND2	55.66
mutserve	PCP	Lost variants	Complex I	MT-ND3	115.61
mutserve	PCP	Lost variants	Complex I	MT-ND4	171.65
mutserve	PCP	Lost variants	Complex I	MT-ND4L	33.67
mutserve	PCP	Lost variants	Complex I	MT-ND5	98.97
mutserve	PCP	Lost variants	Complex I	MT-ND6	51.28
mutserve	PCP	Lost variants	Complex III	MT-CYB	39.15
mutserve	PCP	Lost variants	Complex IV	MT-CO1	50.15
mutserve	PCP	Lost variants	Complex IV	MT-CO2	29.24
mutserve	PCP	Lost variants	Complex IV	MT-CO3	45.98
mutserve	PCP	Lost variants	Complex V	MT-ATP6	39.90
mutserve	PCP	Lost variants	Complex V	MT-ATP8	112.72
mutserve	PCP	Lost variants	D-loop	D-loop	279.26
mutserve	PCP	Lost variants	Other	Other	114.94
mutserve	PCP	Lost variants	rRNA	MT-RNR1	32.15
mutserve	PCP	Lost variants	rRNA	MT-RNR2	34.68
mutserve	PCP	Lost variants	tRNA	MT-TE	144.93
mutserve	PCP	Lost variants	tRNA	MT-TF	328.64
mutserve	PCP	Lost variants	tRNA	MT-TL2	140.85
mutserve	PCP	Lost variants	tRNA	MT-TN	136.99
mutserve	PCP	Lost variants	tRNA	MT-TR	153.85
mutserve	PCP	Lost variants	tRNA	MT-TS2	169.49
mutserve	PCP	Lost variants	tRNA	MT-TT	151.52
mutserve	PCP	Novel variants	Complex IV	MT-CO1	6.49
mutserve	PCP	Novel variants	Complex IV	MT-CO2	14.62
mutserve	PCP	Novel variants	Complex V	MT-ATP6	15.75
mutserve	PCP	Novel variants	Other	Other	114.94
mutserve	PCP	Novel variants	rRNA	MT-RNR2	6.42
mutserve	PCP	Primary novel variants	tRNA	MT-TI	144.93
mutserve	TSS	Found variants	Complex I	MT-ND1	19.43
mutserve	TSS	Found variants	Complex I	MT-ND2	24.95
mutserve	TSS	Found variants	Complex I	MT-ND4	27.85

VCM	Pipeline	Macro variant description	Macro region	Region	Relative burden
mutserve	TSS	Found variants	Complex I	MT-ND5	9.93
mutserve	TSS	Found variants	Complex I	MT-ND6	22.51
mutserve	TSS	Found variants	Complex III	MT-CYB	35.06
mutserve	TSS	Found variants	Complex IV	MT-CO1	16.51
mutserve	TSS	Found variants	Complex V	MT-ATP6	43.04
mutserve	TSS	Found variants	D-loop	D-loop	72.49
mutserve	TSS	Found variants	rRNA	MT-RNR1	28.65
mutserve	TSS	Found variants	rRNA	MT-RNR2	18.10
mutserve	TSS	Found variants	tRNA	MT-TE	144.93
mutserve	TSS	Found variants	tRNA	MT-TL2	140.85
mutserve	TSS	Found variants	tRNA	MT-TR	153.85
mutserve	TSS	Found variants	tRNA	MT-TT	151.52
mutserve	TSS	Lost variants	Complex I	MT-ND1	130.40
mutserve	TSS	Lost variants	Complex I	MT-ND2	61.42
mutserve	TSS	Lost variants	Complex I	MT-ND3	115.61
mutserve	TSS	Lost variants	Complex I	MT-ND4	169.22
mutserve	TSS	Lost variants	Complex I	MT-ND4L	33.67
mutserve	TSS	Lost variants	Complex I	MT-ND5	105.96
mutserve	TSS	Lost variants	Complex I	MT-ND6	50.34
mutserve	TSS	Lost variants	Complex III	MT-CYB	43.82
mutserve	TSS	Lost variants	Complex IV	MT-CO1	54.91
mutserve	TSS	Lost variants	Complex IV	MT-CO2	29.24
mutserve	TSS	Lost variants	Complex IV	MT-CO3	46.83
mutserve	TSS	Lost variants	Complex V	MT-ATP6	46.19
mutserve	TSS	Lost variants	Complex V	MT-ATP8	112.72
mutserve	TSS	Lost variants	D-loop	D-loop	292.93
mutserve	TSS	Lost variants	Other	Other	114.94
mutserve	TSS	Lost variants	rRNA	MT-RNR1	34.24
mutserve	TSS	Lost variants	rRNA	MT-RNR2	35.97
mutserve	TSS	Lost variants	tRNA	MT-TE	144.93
mutserve	TSS	Lost variants	tRNA	MT-TF	328.64
mutserve	TSS	Lost variants	tRNA	MT-TL2	140.85
mutserve	TSS	Lost variants	tRNA	MT-TN	136.99
mutserve	TSS	Lost variants	tRNA	MT-TR	153.85
mutserve	TSS	Lost variants	tRNA	MT-TS2	169.49
mutserve	TSS	Lost variants	tRNA	MT-TT	151.52
mutserve	TSS	Novel variants	Complex I	MT-ND4	7.29
mutserve	TSS	Novel variants	Complex III	MT-CYB	8.76
mutserve	TSS	Novel variants	Complex IV	MT-CO2	14.62
mutserve	TSS	Novel variants	D-loop	D-loop	8.91
mutserve	TSS	Primary novel variants	Complex I	MT-ND4	7.29
mutserve	TSS	Primary novel variants	D-loop	D-loop	8.91
varscan	PCP	Found variants	Complex I	MT-ND1	27.46
varscan	PCP	Found variants	Complex I	MT-ND2	30.71
varscan	PCP	Found variants	Complex I	MT-ND4	25.53
varscan	PCP	Found variants	Complex I	MT-ND5	16.92
varscan	PCP	Found variants	Complex I	MT-ND6	23.81
varscan	PCP	Found variants	Complex III	MT-CYB	37.98
varscan	PCP	Found variants	Complex IV	MT-CO1	21.08

VCM	Pipeline	Macro variant description	Macro region	Region	Relative burden
varscan	PCP	Found variants	Complex IV	MT-CO3	12.77
varscan	PCP	Found variants	Complex V	MT-ATP6	48.29
varscan	PCP	Found variants	D-loop	D-loop	74.87
varscan	PCP	Found variants	rRNA	MT-RNR1	30.75
varscan	PCP	Found variants	rRNA	MT-RNR2	17.66
varscan	PCP	Found variants	tRNA	MT-TE	144.93
varscan	PCP	Found variants	tRNA	MT-TL2	140.85
varscan	PCP	Found variants	tRNA	MT-TN	136.99
varscan	PCP	Found variants	tRNA	MT-TR	153.85
varscan	PCP	Found variants	tRNA	MT-TT	151.52
varscan	PCP	Lost variants	Complex I	MT-ND1	51.60
varscan	PCP	Lost variants	Complex I	MT-ND2	36.47
varscan	PCP	Lost variants	Complex I	MT-ND3	77.07
varscan	PCP	Lost variants	Complex I	MT-ND4	71.97
varscan	PCP	Lost variants	Complex I	MT-ND4L	33.67
varscan	PCP	Lost variants	Complex I	MT-ND5	45.62
varscan	PCP	Lost variants	Complex I	MT-ND6	36.63
varscan	PCP	Lost variants	Complex III	MT-CYB	26.29
varscan	PCP	Lost variants	Complex IV	MT-CO1	32.86
varscan	PCP	Lost variants	Complex IV	MT-CO2	19.49
varscan	PCP	Lost variants	Complex IV	MT-CO3	33.21
varscan	PCP	Lost variants	Complex V	MT-ATP6	40.94
varscan	PCP	Lost variants	Complex V	MT-ATP8	96.62
varscan	PCP	Lost variants	D-loop	D-loop	168.75
varscan	PCP	Lost variants	rRNA	MT-RNR1	18.17
varscan	PCP	Lost variants	rRNA	MT-RNR2	35.11
varscan	PCP	Lost variants	tRNA	MT-TE	144.93
varscan	PCP	Lost variants	tRNA	MT-TL2	140.85
varscan	PCP	Lost variants	tRNA	MT-TN	136.99
varscan	PCP	Lost variants	tRNA	MT-TR	153.85
varscan	PCP	Lost variants	tRNA	MT-TT	151.52
varscan	PCP	Novel variants	Complex IV	MT-CO2	14.62
varscan	PCP	Novel variants	Complex V	MT-ATP6	15.75
varscan	PCP	Novel variants	Other	Other	114.94
varscan	PCP	Primary novel variants	tRNA	MT-TI	144.93
varscan	TSS	Found variants	Complex I	MT-ND1	19.43
varscan	TSS	Found variants	Complex I	MT-ND2	24.95
varscan	TSS	Found variants	Complex I	MT-ND4	24.53
varscan	TSS	Found variants	Complex I	MT-ND5	9.93
varscan	TSS	Found variants	Complex I	MT-ND6	22.51
varscan	TSS	Found variants	Complex III	MT-CYB	35.06
varscan	TSS	Found variants	Complex IV	MT-CO1	16.51
varscan	TSS	Found variants	Complex V	MT-ATP6	43.04
varscan	TSS	Found variants	D-loop	D-loop	72.49
varscan	TSS	Found variants	rRNA	MT-RNR1	28.65
varscan	TSS	Found variants	rRNA	MT-RNR2	18.10
varscan	TSS	Found variants	tRNA	MT-TE	144.93
varscan	TSS	Found variants	tRNA	MT-TL2	140.85
varscan	TSS	Found variants	tRNA	MT-TR	153.85

VCM	Pipeline	Macro variant description	Macro region	Region	Relative burden
varscan	TSS	Found variants	tRNA	MT-TT	151.52
varscan	TSS	Lost variants	Complex I	MT-ND1	57.18
varscan	TSS	Lost variants	Complex I	MT-ND2	42.23
varscan	TSS	Lost variants	Complex I	MT-ND3	77.07
varscan	TSS	Lost variants	Complex I	MT-ND4	74.40
varscan	TSS	Lost variants	Complex I	MT-ND4L	33.67
varscan	TSS	Lost variants	Complex I	MT-ND5	52.61
varscan	TSS	Lost variants	Complex I	MT-ND6	36.73
varscan	TSS	Lost variants	Complex III	MT-CYB	29.21
varscan	TSS	Lost variants	Complex IV	MT-CO1	37.61
varscan	TSS	Lost variants	Complex IV	MT-CO2	19.49
varscan	TSS	Lost variants	Complex IV	MT-CO3	34.06
varscan	TSS	Lost variants	Complex V	MT-ATP6	46.19
varscan	TSS	Lost variants	Complex V	MT-ATP8	96.62
varscan	TSS	Lost variants	D-loop	D-loop	171.12
varscan	TSS	Lost variants	rRNA	MT-RNR1	20.27
varscan	TSS	Lost variants	rRNA	MT-RNR2	35.97
varscan	TSS	Lost variants	tRNA	MT-TE	144.93
varscan	TSS	Lost variants	tRNA	MT-TL2	140.85
varscan	TSS	Lost variants	tRNA	MT-TN	136.99
varscan	TSS	Lost variants	tRNA	MT-TR	153.85
varscan	TSS	Lost variants	tRNA	MT-TT	151.52
varscan	TSS	Novel variants	Complex I	MT-ND4	7.29
varscan	TSS	Novel variants	Complex III	MT-CYB	8.76
varscan	TSS	Novel variants	Complex IV	MT-CO2	14.62
varscan	TSS	Novel variants	D-loop	D-loop	8.91
varscan	TSS	Primary novel variants	Complex I	MT-ND4	7.29
varscan	TSS	Primary novel variants	D-loop	D-loop	8.91

Table S16 - Mixture analysis: Relative burden of different macro variants in different mtDNA regions, depending on the VCM chosen. Macro regions only

VCM	Pipeline	Macro variant description	Macro region	Relative burden
freebayes	PCP	Found variants	Complex I	17.64
freebayes	PCP	Found variants	Complex III	39.73
freebayes	PCP	Found variants	Complex IV	11.08
freebayes	PCP	Found variants	Complex V	25.34
freebayes	PCP	Found variants	D-loop	66.55
freebayes	PCP	Found variants	rRNA	20.44
freebayes	PCP	Found variants	tRNA	15.04
freebayes	PCP	Lost variants	Complex I	131.46
freebayes	PCP	Lost variants	Complex III	53.75
freebayes	PCP	Lost variants	Complex IV	40.99
freebayes	PCP	Lost variants	Complex V	93.43
freebayes	PCP	Lost variants	D-loop	251.34
freebayes	PCP	Lost variants	rRNA	40.62
freebayes	PCP	Lost variants	tRNA	29.20
freebayes	PCP	Novel variants	Complex I	2.00
freebayes	PCP	Novel variants	Complex IV	3.32
freebayes	PCP	Novel variants	Complex V	12.87
freebayes	PCP	Novel variants	tRNA	6.64
freebayes	PCP	Primary novel variants	Complex V	11.88
freebayes	PCP	Primary novel variants	tRNA	9.01
freebayes	TSS	Found variants	Complex I	13.55
freebayes	TSS	Found variants	Complex III	35.06
freebayes	TSS	Found variants	Complex IV	8.46
freebayes	TSS	Found variants	Complex V	26.99
freebayes	TSS	Found variants	D-loop	65.36
freebayes	TSS	Found variants	rRNA	19.12
freebayes	TSS	Found variants	tRNA	15.68
freebayes	TSS	Lost variants	Complex I	135.56
freebayes	TSS	Lost variants	Complex III	58.43
freebayes	TSS	Lost variants	Complex IV	43.65
freebayes	TSS	Lost variants	Complex V	91.05
freebayes	TSS	Lost variants	D-loop	252.53
freebayes	TSS	Lost variants	rRNA	41.95
freebayes	TSS	Lost variants	tRNA	32.74
freebayes	TSS	Novel variants	Complex I	1.58
freebayes	TSS	Novel variants	Complex III	8.76
freebayes	TSS	Novel variants	Complex IV	3.32
freebayes	TSS	Novel variants	Complex V	19.00
freebayes	TSS	Novel variants	D-loop	19.10
freebayes	TSS	Primary novel variants	Complex I	1.58
freebayes	TSS	Primary novel variants	Complex V	11.88
freebayes	TSS	Primary novel variants	D-loop	16.84
mutserve	PCP	Found variants	Complex I	18.06
mutserve	PCP	Found variants	Complex III	39.73
mutserve	PCP	Found variants	Complex IV	11.08
mutserve	PCP	Found variants	Complex V	37.21
mutserve	PCP	Found variants	D-loop	86.16
mutserve	PCP	Found variants	rRNA	20.71

VCM	Pipeline	Macro variant description	Macro region	Relative burden
mutserve	PCP	Found variants	tRNA	15.04
mutserve	PCP	Lost variants	Complex I	104.27
mutserve	PCP	Lost variants	Complex III	39.15
mutserve	PCP	Lost variants	Complex IV	44.31
mutserve	PCP	Lost variants	Complex V	57.80
mutserve	PCP	Lost variants	D-loop	279.26
mutserve	PCP	Lost variants	Other	114.94
mutserve	PCP	Lost variants	rRNA	33.72
mutserve	PCP	Lost variants	tRNA	26.99
mutserve	PCP	Novel variants	Complex IV	3.32
mutserve	PCP	Novel variants	Complex V	11.88
mutserve	PCP	Novel variants	Other	114.94
mutserve	PCP	Novel variants	rRNA	3.98
mutserve	PCP	Primary novel variants	tRNA	6.64
mutserve	TSS	Found variants	Complex I	14.07
mutserve	TSS	Found variants	Complex III	35.06
mutserve	TSS	Found variants	Complex IV	8.46
mutserve	TSS	Found variants	Complex V	32.46
mutserve	TSS	Found variants	D-loop	72.49
mutserve	TSS	Found variants	rRNA	19.12
mutserve	TSS	Found variants	tRNA	15.68
mutserve	TSS	Lost variants	Complex I	107.73
mutserve	TSS	Lost variants	Complex III	43.82
mutserve	TSS	Lost variants	Complex IV	46.97
mutserve	TSS	Lost variants	Complex V	62.55
mutserve	TSS	Lost variants	D-loop	292.93
mutserve	TSS	Lost variants	Other	114.94
mutserve	TSS	Lost variants	rRNA	35.31
mutserve	TSS	Lost variants	tRNA	30.52
mutserve	TSS	Novel variants	Complex I	1.58
mutserve	TSS	Novel variants	Complex III	8.76
mutserve	TSS	Novel variants	Complex IV	3.32
mutserve	TSS	Novel variants	D-loop	8.91
mutserve	TSS	Primary novel variants	Complex I	1.58
mutserve	TSS	Primary novel variants	D-loop	8.91
varscan	PCP	Found variants	Complex I	18.06
varscan	PCP	Found variants	Complex III	37.98
varscan	PCP	Found variants	Complex IV	11.08
varscan	PCP	Found variants	Complex V	36.42
varscan	PCP	Found variants	D-loop	74.87
varscan	PCP	Found variants	rRNA	20.44
varscan	PCP	Found variants	tRNA	14.60
varscan	PCP	Lost variants	Complex I	50.19
varscan	PCP	Lost variants	Complex III	26.29
varscan	PCP	Lost variants	Complex IV	29.91
varscan	PCP	Lost variants	Complex V	54.63
varscan	PCP	Lost variants	D-loop	168.75
varscan	PCP	Lost variants	rRNA	28.67
varscan	PCP	Lost variants	tRNA	8.68

VCM	Pipeline	Macro variant description	Macro region	Relative burden
varscan	PCP	Novel variants	Complex IV	3.32
varscan	PCP	Novel variants	Complex V	11.88
varscan	PCP	Novel variants	Other	114.94
varscan	PCP	Primary novel variants	tRNA	6.64
varscan	TSS	Found variants	Complex I	13.55
varscan	TSS	Found variants	Complex III	35.06
varscan	TSS	Found variants	Complex IV	8.46
varscan	TSS	Found variants	Complex V	32.46
varscan	TSS	Found variants	D-loop	72.49
varscan	TSS	Found variants	rRNA	19.12
varscan	TSS	Found variants	tRNA	15.68
varscan	TSS	Lost variants	Complex I	54.71
varscan	TSS	Lost variants	Complex III	29.21
varscan	TSS	Lost variants	Complex IV	32.57
varscan	TSS	Lost variants	Complex V	58.59
varscan	TSS	Lost variants	D-loop	171.12
varscan	TSS	Lost variants	rRNA	30.00
varscan	TSS	Lost variants	tRNA	11.38
varscan	TSS	Novel variants	Complex I	1.58
varscan	TSS	Novel variants	Complex III	8.76
varscan	TSS	Novel variants	Complex IV	3.32
varscan	TSS	Novel variants	D-loop	8.91
varscan	TSS	Primary novel variants	Complex I	1.58
varscan	TSS	Primary novel variants	D-loop	8.91

Table S17 - Mixture analysis: Paired comparison of mean normalized coverage per sample in different macro variants, depending on the VCM chosen. Pairwise t-test with FDR

VCM	Pipeline	Comparison	Means	p-value
freebayes	PCP	Lost variants - Found variants	0.89 ~ 1.06	2.69E-05
freebayes	PCP	Lost variants - Novel variants	0.89 ~ 0.22	8.55E-25
freebayes	PCP	Novel variants - Found variants	0.22 ~ 1.06	6.67E-29
freebayes	PCP	Primary novel variants - Found variants	0.34 ~ 1.06	7.20E-26
freebayes	PCP	Primary novel variants - Lost variants	0.34 ~ 0.89	6.18E-21
freebayes	PCP	Primary novel variants - Novel variants	0.34 ~ 0.22	1.54E-03
freebayes	TSS	Lost variants - Found variants	0.90 ~ 1.02	3.69E-02
freebayes	TSS	Lost variants - Novel variants	0.90 ~ 0.52	3.30E-08
freebayes	TSS	Novel variants - Found variants	0.52 ~ 1.02	3.13E-11
freebayes	TSS	Primary novel variants - Found variants	0.36 ~ 1.02	7.54E-13
freebayes	TSS	Primary novel variants - Lost variants	0.36 ~ 0.90	1.54E-10
freebayes	TSS	Primary novel variants - Novel variants	0.36 ~ 0.52	2.06E-02
mutserve	PCP	Lost variants - Found variants	0.91 ~ 1.04	1.06E-04
mutserve	PCP	Lost variants - Novel variants	0.91 ~ 0.41	3.92E-18
mutserve	PCP	Novel variants - Found variants	0.41 ~ 1.04	1.64E-21
mutserve	PCP	Primary novel variants - Found variants	0.54 ~ 1.04	1.72E-18
mutserve	PCP	Primary novel variants - Lost variants	0.54 ~ 0.91	3.59E-14
mutserve	PCP	Primary novel variants - Novel variants	0.54 ~ 0.41	1.36E-03
mutserve	TSS	Lost variants - Found variants	0.91 ~ 1.00	1.70E-01
mutserve	TSS	Lost variants - Novel variants	0.91 ~ 0.49	1.43E-07
mutserve	TSS	Novel variants - Found variants	0.49 ~ 1.00	1.68E-09
mutserve	TSS	Primary novel variants - Found variants	0.19 ~ 1.00	1.15E-13
mutserve	TSS	Primary novel variants - Lost variants	0.19 ~ 0.91	2.89E-12
mutserve	TSS	Primary novel variants - Novel variants	0.19 ~ 0.49	4.23E-04
varscan	PCP	Lost variants - Found variants	0.98 ~ 1.06	7.37E-03
varscan	PCP	Lost variants - Novel variants	0.98 ~ 0.37	1.89E-22
varscan	PCP	Novel variants - Found variants	0.37 ~ 1.06	2.73E-24
varscan	PCP	Primary novel variants - Found variants	0.54 ~ 1.06	1.19E-20
varscan	PCP	Primary novel variants - Lost variants	0.54 ~ 0.98	4.27E-18
varscan	PCP	Primary novel variants - Novel variants	0.54 ~ 0.37	4.21E-05
varscan	TSS	Lost variants - Found variants	0.99 ~ 1.01	6.26E-01
varscan	TSS	Lost variants - Novel variants	0.99 ~ 0.31	1.30E-21
varscan	TSS	Novel variants - Found variants	0.31 ~ 1.01	4.95E-22
varscan	TSS	Primary novel variants - Found variants	0.17 ~ 1.01	2.62E-22
varscan	TSS	Primary novel variants - Lost variants	0.17 ~ 0.99	3.75E-22
varscan	TSS	Primary novel variants - Novel variants	0.17 ~ 0.31	5.58E-03

Table S18 - Mixture analysis: Paired comparison of mean coverage ratios per sample in different macro variants, depending on the VCM chosen. Pairwise t-test with FDR

VCM	Pipeline	Comparison	Means	p-value
freebayes	PCP	Lost variants - Found variants	17.17 ~ 4.09	1.50E-16
freebayes	PCP	Lost variants - Novel variants	17.17 ~ 4.95	5.33E-14
freebayes	PCP	Novel variants - Found variants	4.95 ~ 4.09	4.46E-01
freebayes	PCP	Primary novel variants - Found variants	1.04 ~ 4.09	1.13E-02
freebayes	PCP	Primary novel variants - Lost variants	1.04 ~ 17.17	8.00E-18
freebayes	PCP	Primary novel variants - Novel variants	1.04 ~ 4.95	4.02E-03
freebayes	TSS	Lost variants - Found variants	16.10 ~ 1.59	1.76E-34
freebayes	TSS	Lost variants - Novel variants	16.10 ~ 2.28	9.12E-34
freebayes	TSS	Novel variants - Found variants	2.28 ~ 1.59	1.26E-01
freebayes	TSS	Primary novel variants - Found variants	3.39 ~ 1.59	1.52E-03
freebayes	TSS	Primary novel variants - Lost variants	3.39 ~ 16.10	2.64E-29
freebayes	TSS	Primary novel variants - Novel variants	3.39 ~ 2.28	4.41E-02
mutserve	PCP	Lost variants - Found variants	1.78 ~ 2.46	8.35E-01
mutserve	PCP	Lost variants - Novel variants	1.78 ~ 12.10	3.82E-02
mutserve	PCP	Novel variants - Found variants	12.10 ~ 2.46	3.82E-02
mutserve	PCP	Primary novel variants - Found variants	3.78 ~ 2.46	8.35E-01
mutserve	PCP	Primary novel variants - Lost variants	3.78 ~ 1.78	8.35E-01
mutserve	PCP	Primary novel variants - Novel variants	3.78 ~ 12.10	9.18E-02
mutserve	TSS	Lost variants - Found variants	1.55 ~ 1.58	8.69E-01
mutserve	TSS	Lost variants - Novel variants	1.55 ~ 2.18	1.26E-04
mutserve	TSS	Novel variants - Found variants	2.18 ~ 1.58	1.70E-04
mutserve	TSS	Primary novel variants - Found variants	3.57 ~ 1.58	5.98E-16
mutserve	TSS	Primary novel variants - Lost variants	3.57 ~ 1.55	5.98E-16
mutserve	TSS	Primary novel variants - Novel variants	3.57 ~ 2.18	1.41E-10
varscan	PCP	Lost variants - Found variants	1.45 ~ 1.50	9.73E-01
varscan	PCP	Lost variants - Novel variants	1.45 ~ 5.33	5.88E-02
varscan	PCP	Novel variants - Found variants	5.33 ~ 1.50	5.88E-02
varscan	PCP	Primary novel variants - Found variants	3.81 ~ 1.50	2.08E-01
varscan	PCP	Primary novel variants - Lost variants	3.81 ~ 1.45	2.08E-01
varscan	PCP	Primary novel variants - Novel variants	3.81 ~ 5.33	4.59E-01
varscan	TSS	Lost variants - Found variants	1.45 ~ 1.57	2.91E-01
varscan	TSS	Lost variants - Novel variants	1.45 ~ 2.35	4.94E-10
varscan	TSS	Novel variants - Found variants	2.35 ~ 1.57	1.83E-08
varscan	TSS	Primary novel variants - Found variants	3.62 ~ 1.57	4.64E-20
varscan	TSS	Primary novel variants - Lost variants	3.62 ~ 1.45	8.02E-21
varscan	TSS	Primary novel variants - Novel variants	3.62 ~ 2.35	1.78E-12

Table S19 - Mixture analysis: Paired comparison of NUMT rates per sample in different macro variants, depending on the VCM chosen. Pairwise t-test with FDR

VCM	Pipeline	Comparison	Means	p-value
freebayes	PCP	Lost variants - Found variants	2.27 ~ 3.82	2.12E-04
freebayes	PCP	Novel variants - Found variants	1.87 ~ 3.82	1.07E-05
freebayes	PCP	Novel variants - Lost variants	1.87 ~ 2.27	3.16E-01
freebayes	PCP	Primary novel variants - Found variants	6.32 ~ 3.82	8.70E-08
freebayes	PCP	Primary novel variants - Lost variants	6.32 ~ 2.27	6.49E-14
freebayes	PCP	Primary novel variants - Novel variants	6.32 ~ 1.87	7.60E-15
freebayes	TSS	Lost variants - Found variants	2.25 ~ 4.19	1.38E-02
freebayes	TSS	Novel variants - Found variants	3.91 ~ 4.19	6.64E-01
freebayes	TSS	Novel variants - Lost variants	3.91 ~ 2.25	2.92E-02
freebayes	TSS	Primary novel variants - Found variants	5.14 ~ 4.19	2.59E-01
freebayes	TSS	Primary novel variants - Lost variants	5.14 ~ 2.25	2.20E-03
freebayes	TSS	Primary novel variants - Novel variants	5.14 ~ 3.91	1.64E-01
mutserve	PCP	Lost variants - Found variants	2.25 ~ 4.02	8.02E-15
mutserve	PCP	Novel variants - Found variants	3.01 ~ 4.02	1.13E-06
mutserve	PCP	Novel variants - Lost variants	3.01 ~ 2.25	9.41E-05
mutserve	PCP	Primary novel variants - Found variants	10.50 ~ 4.02	2.43E-35
mutserve	PCP	Primary novel variants - Lost variants	10.50 ~ 2.25	1.77E-39
mutserve	PCP	Primary novel variants - Novel variants	10.50 ~ 3.01	1.14E-35
mutserve	TSS	Lost variants - Found variants	2.30 ~ 4.21	6.02E-10
mutserve	TSS	Novel variants - Found variants	1.97 ~ 4.21	3.54E-11
mutserve	TSS	Novel variants - Lost variants	1.97 ~ 2.30	1.93E-01
mutserve	TSS	Primary novel variants - Found variants	3.67 ~ 4.21	6.39E-02
mutserve	TSS	Primary novel variants - Lost variants	3.67 ~ 2.30	1.34E-05
mutserve	TSS	Primary novel variants - Novel variants	3.67 ~ 1.97	5.27E-07
varscan	PCP	Lost variants - Found variants	2.45 ~ 4.09	1.50E-19
varscan	PCP	Novel variants - Found variants	2.97 ~ 4.09	8.06E-12
varscan	PCP	Novel variants - Lost variants	2.97 ~ 2.45	9.69E-05
varscan	PCP	Primary novel variants - Found variants	10.50 ~ 4.09	2.19E-42
varscan	PCP	Primary novel variants - Lost variants	10.50 ~ 2.45	2.60E-46
varscan	PCP	Primary novel variants - Novel variants	10.50 ~ 2.97	4.91E-43
varscan	TSS	Lost variants - Found variants	2.49 ~ 4.24	7.52E-13
varscan	TSS	Novel variants - Found variants	1.57 ~ 4.24	3.79E-19
varscan	TSS	Novel variants - Lost variants	1.57 ~ 2.49	7.02E-06
varscan	TSS	Primary novel variants - Found variants	3.67 ~ 4.24	7.80E-03
varscan	TSS	Primary novel variants - Lost variants	3.67 ~ 2.49	1.06E-06
varscan	TSS	Primary novel variants - Novel variants	3.67 ~ 1.57	3.94E-13

Table S20 - Mixture analysis: Paired comparison of mean prevalence of LCR variants per sample in different macro variants, depending on the VCM chosen. Pairwise t-test with FDR

VCM	Pipeline	Comparison	Means	p-value
freebayes	PCP	Lost variants - Found variants	38.19% ~ 7.38%	5.85E-35
freebayes	PCP	Novel variants - Found variants	0.00% ~ 7.38%	4.38E-09
freebayes	PCP	Novel variants - Lost variants	0.00% ~ 38.19%	3.42E-39
freebayes	PCP	Primary novel variants - Found variants	0.00% ~ 7.38%	4.38E-09
freebayes	PCP	Primary novel variants - Lost variants	0.00% ~ 38.19%	3.42E-39
freebayes	PCP	Primary novel variants - Novel variants	0.00% ~ 0.00%	1.00E+00
freebayes	TSS	Lost variants - Found variants	37.67% ~ 5.23%	1.14E-11
freebayes	TSS	Novel variants - Found variants	1.67% ~ 5.23%	3.34E-01
freebayes	TSS	Novel variants - Lost variants	1.67% ~ 37.67%	7.97E-13
freebayes	TSS	Primary novel variants - Found variants	45.37% ~ 5.23%	1.69E-12
freebayes	TSS	Primary novel variants - Lost variants	45.37% ~ 37.67%	8.87E-02
freebayes	TSS	Primary novel variants - Novel variants	45.37% ~ 1.67%	2.93E-13
mutserve	PCP	Lost variants - Found variants	38.18% ~ 4.11%	4.77E-35
mutserve	PCP	Novel variants - Found variants	0.00% ~ 4.11%	3.71E-04
mutserve	PCP	Novel variants - Lost variants	0.00% ~ 38.18%	1.17E-34
mutserve	PCP	Primary novel variants - Found variants	0.00% ~ 4.11%	3.11E-04
mutserve	PCP	Primary novel variants - Lost variants	0.00% ~ 38.18%	4.77E-35
mutserve	PCP	Primary novel variants - Novel variants	0.00% ~ 0.00%	1.00E+00
mutserve	TSS	Lost variants - Found variants	36.41% ~ 4.85%	2.32E-09
mutserve	TSS	Novel variants - Found variants	3.85% ~ 4.85%	8.21E-01
mutserve	TSS	Novel variants - Lost variants	3.85% ~ 36.41%	2.32E-09
mutserve	TSS	Primary novel variants - Found variants	88.89% ~ 4.85%	2.67E-21
mutserve	TSS	Primary novel variants - Lost variants	88.89% ~ 36.41%	5.52E-14
mutserve	TSS	Primary novel variants - Novel variants	88.89% ~ 3.85%	2.67E-21
varscan	PCP	Lost variants - Found variants	31.02% ~ 1.38%	2.25E-32
varscan	PCP	Novel variants - Found variants	0.00% ~ 1.38%	2.29E-01
varscan	PCP	Novel variants - Lost variants	0.00% ~ 31.02%	5.17E-31
varscan	PCP	Primary novel variants - Found variants	0.00% ~ 1.38%	2.29E-01
varscan	PCP	Primary novel variants - Lost variants	0.00% ~ 31.02%	1.94E-31
varscan	PCP	Primary novel variants - Novel variants	0.00% ~ 0.00%	1.00E+00
varscan	TSS	Lost variants - Found variants	27.98% ~ 4.85%	1.43E-06
varscan	TSS	Novel variants - Found variants	3.33% ~ 4.85%	7.18E-01
varscan	TSS	Novel variants - Lost variants	3.33% ~ 27.98%	4.92E-07
varscan	TSS	Primary novel variants - Found variants	88.89% ~ 4.85%	2.31E-22
varscan	TSS	Primary novel variants - Lost variants	88.89% ~ 27.98%	7.76E-17
varscan	TSS	Primary novel variants - Novel variants	88.89% ~ 3.33%	2.13E-22

Table S21 - Mixture analysis: Paired comparison of mean distance to the amplicon's edge per sample in different macro variants, depending on the VCM chosen. Pairwise t-test with FDR

VCM	Pipeline	Comparison	Means	p-value
freebayes	PCP	Lost variants - Found variants	19.00 ~ 25.21	6.10E-02
freebayes	PCP	Novel variants - Found variants	25.64 ~ 25.21	8.91E-01
freebayes	PCP	Novel variants - Lost variants	25.64 ~ 19.00	6.05E-02
freebayes	PCP	Primary novel variants - Found variants	9.79 ~ 25.21	2.99E-05
freebayes	PCP	Primary novel variants - Lost variants	9.79 ~ 19.00	1.04E-02
freebayes	PCP	Primary novel variants - Novel variants	9.79 ~ 25.64	2.99E-05
freebayes	TSS	Lost variants - Found variants	19.31 ~ 24.88	2.79E-03
freebayes	TSS	Novel variants - Found variants	30.42 ~ 24.88	2.79E-03
freebayes	TSS	Novel variants - Lost variants	30.42 ~ 19.31	1.53E-07
freebayes	TSS	Primary novel variants - Found variants	24.03 ~ 24.88	6.63E-01
freebayes	TSS	Primary novel variants - Lost variants	24.03 ~ 19.31	2.26E-02
freebayes	TSS	Primary novel variants - Novel variants	24.03 ~ 30.42	2.79E-03
mutserve	PCP	Lost variants - Found variants	17.78 ~ 24.72	6.41E-04
mutserve	PCP	Novel variants - Found variants	9.61 ~ 24.72	1.79E-08
mutserve	PCP	Novel variants - Lost variants	9.61 ~ 17.78	6.33E-04
mutserve	PCP	Primary novel variants - Found variants	2.00 ~ 24.72	1.78E-13
mutserve	PCP	Primary novel variants - Lost variants	2.00 ~ 17.78	4.05E-09
mutserve	PCP	Primary novel variants - Novel variants	2.00 ~ 9.61	2.16E-03
mutserve	TSS	Lost variants - Found variants	18.10 ~ 25.09	2.89E-02
mutserve	TSS	Novel variants - Found variants	30.09 ~ 25.09	1.22E-01
mutserve	TSS	Novel variants - Lost variants	30.09 ~ 18.10	6.46E-04
mutserve	TSS	Primary novel variants - Found variants	13.67 ~ 25.09	2.52E-03
mutserve	TSS	Primary novel variants - Lost variants	13.67 ~ 18.10	1.90E-01
mutserve	TSS	Primary novel variants - Novel variants	13.67 ~ 30.09	9.79E-05
varscan	PCP	Lost variants - Found variants	19.25 ~ 25.59	1.74E-03
varscan	PCP	Novel variants - Found variants	7.61 ~ 25.59	2.90E-10
varscan	PCP	Novel variants - Lost variants	7.61 ~ 19.25	3.65E-06
varscan	PCP	Primary novel variants - Found variants	2.00 ~ 25.59	6.01E-14
varscan	PCP	Primary novel variants - Lost variants	2.00 ~ 19.25	2.90E-10
varscan	PCP	Primary novel variants - Novel variants	2.00 ~ 7.61	2.13E-02
varscan	TSS	Lost variants - Found variants	19.80 ~ 25.09	4.51E-02
varscan	TSS	Novel variants - Found variants	30.94 ~ 25.09	4.08E-02
varscan	TSS	Novel variants - Lost variants	30.94 ~ 19.80	2.14E-04
varscan	TSS	Primary novel variants - Found variants	13.67 ~ 25.09	6.86E-04
varscan	TSS	Primary novel variants - Lost variants	13.67 ~ 19.80	4.51E-02
varscan	TSS	Primary novel variants - Novel variants	13.67 ~ 30.94	2.55E-06

Table S22 - Performance analysis: Paired difference between different performance indicators for Grade A/B variants, depending on the VCM chosen. Bootstrap with FDR for the primary analysis and paired t-test with FDR for the mixture analysis

VCM	Dataset	Grade	Performance indicator	Mean TSS-PCP paired difference [95% CI]	p-value	p-value (adj)
freebayes	primary	A	Sensitivity	1.45% [0.00% - 4.35%]	5.15E-01	5.80E-01
			Specificity	-0.01% [-0.02% - 0.00%]	7.63E-02	1.57E-01
			Precision	-5.20% [-12.39% - 0.00%]	7.29E-02	1.57E-01
			F_1 score	-2.23% [-6.96% - 0.26%]	5.13E-01	5.80E-01
		B	Sensitivity	-5.82% [-8.62% - -3.70%]	0.00E+00	0.00E+00
			Specificity	-0.01% [-0.02% - 0.00%]	7.28E-02	1.57E-01
			Precision	-4.39% [-10.22% - 0.00%]	7.85E-02	1.57E-01
			F_1 score	-5.99% [-7.07% - -4.05%]	0.00E+00	0.00E+00
	mixture	A	Sensitivity	-7.64% [-21.53% - 6.25%]	2.58E-01	2.58E-01
			Specificity	-0.01% [-0.01% - 0.00%]	3.89E-03	2.39E-02
			Precision	-9.39% [-17.58% - -1.19%]	2.77E-02	5.02E-02
			F_1 score	-7.81% [-19.01% - 3.39%]	1.57E-01	1.98E-01
		B	Sensitivity	-6.39% [-11.86% - -0.93%]	2.49E-02	4.98E-02
			Specificity	-0.01% [-0.01% - 0.00%]	3.90E-03	2.39E-02
			Precision	-7.12% [-11.30% - -2.95%]	2.58E-03	2.39E-02
			F_1 score	-7.06% [-12.83% - -1.29%]	2.00E-02	4.81E-02
mutserve	primary	A	Sensitivity	-1.71% [-5.13% - 0.00%]	5.21E-01	5.80E-01
			Specificity	0.00% [-0.01% - 0.01%]	7.37E-01	7.40E-01
			Precision	-1.32% [-6.45% - 2.50%]	5.13E-01	5.80E-01
			F_1 score	-1.57% [-3.33% - 0.00%]	7.44E-02	1.57E-01
		B	Sensitivity	-8.16% [-13.11% - -5.13%]	0.00E+00	0.00E+00
			Specificity	0.00% [-0.01% - 0.01%]	7.40E-01	7.40E-01
			Precision	-1.46% [-6.45% - 2.08%]	5.13E-01	5.80E-01
			F_1 score	-6.76% [-8.24% - -5.71%]	0.00E+00	0.00E+00

VCM	Dataset	Grade	Performance indicator	Mean TSS-PCP paired difference [95% CI]	p-value	p-value (adj)
	mixture	A	Sensitivity	-10.45% [-20.93% - 0.04%]	5.07E-02	7.16E-02
			Specificity	0.00% [-0.01% - 0.00%]	2.50E-01	2.58E-01
			Precision	-4.25% [-8.10% - -0.40%]	3.29E-02	5.02E-02
			F1 score	-7.44% [-15.11% - 0.23%]	5.62E-02	7.50E-02
		B	Sensitivity	-8.84% [-14.66% - -3.02%]	5.71E-03	2.39E-02
			Specificity	0.00% [-0.01% - 0.00%]	2.50E-01	2.58E-01
			Precision	-4.27% [-7.84% - -0.69%]	2.28E-02	4.98E-02
			F1 score	-8.55% [-14.33% - -2.76%]	6.85E-03	2.39E-02
varscan	primary	A	Sensitivity	7.50% [-2.56% - 18.18%]	7.09E-02	1.57E-01
			Specificity	0.00% [-0.01% - 0.00%]	5.25E-01	5.80E-01
			Precision	-2.17% [-6.45% - 0.00%]	7.28E-02	1.57E-01
			F1 score	2.97% [-1.33% - 10.00%]	5.32E-01	5.80E-01
		B	Sensitivity	-2.81% [-11.86% - 3.45%]	5.16E-01	5.80E-01
			Specificity	0.00% [-0.01% - 0.00%]	5.17E-01	5.80E-01
			Precision	-2.27% [-6.45% - 0.00%]	7.43E-02	1.57E-01
			F1 score	-2.38% [-8.00% - 3.54%]	2.98E-01	5.49E-01
	mixture	A	Sensitivity	-6.40% [-16.75% - 3.96%]	2.06E-01	2.36E-01
			Specificity	-0.01% [-0.01% - 0.00%]	1.04E-02	2.78E-02
			Precision	-5.03% [-8.45% - -1.61%]	6.98E-03	2.39E-02
			F1 score	-4.87% [-12.74% - 3.00%]	2.05E-01	2.36E-01
		B	Sensitivity	-7.02% [-13.23% - -0.80%]	2.96E-02	5.02E-02
			Specificity	-0.01% [-0.01% - 0.00%]	1.04E-02	2.78E-02
			Precision	-5.08% [-8.27% - -1.89%]	4.16E-03	2.39E-02
			F1 score	-6.73% [-12.86% - -0.61%]	3.34E-02	5.02E-02

Table S23 - Primary analysis: Distribution of variants with freebayes and varscan

VCM	Variant description		PCP				TSS			
	Broad classification	Detailed classification	*N*	Mean observed VL<br>[Min - Max]	Mean primary VL<br>[Min - Max]	Mean absolute difference in VLs<br>[Min - Max]	*N*	Mean observed VL<br>[Min - Max]	Mean primary VL<br>[Min - Max]	Mean absolute difference in VLs<br>[Min - Max]
freebayes	Grade A variant found	including exome	*43*	99.72%<br>[98.56% - 100.00%]	99.56%<br>[96.99% - 99.99%]	0.48%<br>[0.00% - 2.71%]	*44*	99.41%<br>[98.60% - 100.00%]	99.57%<br>[96.99% - 99.99%]	0.68%<br>[0.03% - 2.64%]
	Grade B variant found	including exome	*5*	26.10%<br>[3.52% - 92.55%]	28.59%<br>[2.57% - 94.75%]	2.88%<br>[0.95% - 7.42%]	*2*	55.65%<br>[19.60% - 91.70%]	58.56%<br>[22.37% - 94.75%]	2.91%<br>[2.77% - 3.05%]
		excluding exome	*7*	7.72%<br>[2.67% - 28.39%]	6.86%<br>[1.12% - 28.31%]	0.87%<br>[0.03% - 2.06%]	*1*	28.30%	28.31%	0.01%
		from highCov and exome	*3*	99.94%<br>[99.83% - 100.00%]	99.97%<br>[99.94% - 99.99%]	0.08%<br>[0.02% - 0.16%]	*3*	99.47%<br>[99.30% - 99.60%]	99.97%<br>[99.94% - 99.99%]	0.51%<br>[0.34% - 0.69%]
		from lowCov and exome	*21*	99.74%<br>[98.46% - 100.00%]	99.30%<br>[95.83% - 100.00%]	0.75%<br>[0.00% - 3.88%]	*21*	99.50%<br>[98.80% - 100.00%]	99.30%<br>[95.83% - 100.00%]	0.87%<br>[0.00% - 4.17%]
	Grade C variant found	from exome	*4*	99.78%<br>[99.56% - 100.00%]	100.00%<br>[100.00% - 100.00%]	0.22%<br>[0.00% - 0.44%]	*5*	99.62%<br>[99.40% - 99.90%]	99.96%<br>[99.79% - 100.00%]	0.38%<br>[0.11% - 0.60%]
	Grade A variant lost	including exome	*1*	-	99.99%	-	-	-	-	-
	Grade B variant lost	including exome	*5*	-	0.83%<br>[0.54% - 1.27%]	-	*8*	-	3.75%<br>[0.54% - 17.37%]	-
		excluding exome	*32*	-	1.19%<br>[0.43% - 5.75%]	-	*38*	-	1.52%<br>[0.43% - 6.13%]	-
		from lowCov and exome	*7*	-	2.03%<br>[0.44% - 4.59%]	-	*7*	-	2.03%<br>[0.44% - 4.59%]	-
	Grade C variant lost	from highCov	*210*	-	0.76%<br>[0.40% - 2.52%]	-	*210*	-	0.76%<br>[0.40% - 2.52%]	-
		from lowCov	*19*	-	0.88%<br>[0.40% - 2.35%]	-	*19*	-	0.88%<br>[0.40% - 2.35%]	-
		from exome	*10*	-	10.54%<br>[0.43% - 99.79%]	-	*9*	-	0.63%<br>[0.43% - 1.04%]	-
	Novel variant	only present in Ion Torrent	*3*	4.57%<br>[2.52% - 8.44%]	-	-	*7*	66.87%<br>[19.30% - 99.90%]	-	-
varscan	Grade A variant found	including exome	*74*	99.75%<br>[98.46% - 100.00%]	99.62%<br>[97.07% - 100.00%]	0.44%<br>[0.00% - 2.78%]	*77*	99.43%<br>[98.20% - 100.00%]	99.62%<br>[97.07% - 100.00%]	0.66%<br>[0.00% - 2.78%]

VCM	Variant description		PCP				TSS			
	Broad classification	Detailed classification	*N*	Mean observed VL [Min - Max]	Mean primary VL [Min - Max]	Mean absolute difference in VLs [Min - Max]	*N*	Mean observed VL [Min - Max]	Mean primary VL [Min - Max]	Mean absolute difference in VLs [Min - Max]
	Grade B variant found	including exome	*5*	26.10% [3.52% - 92.55%]	28.61% [2.58% - 94.80%]	2.89% [0.94% - 7.43%]	*2*	55.65% [19.60% - 91.70%]	58.59% [22.38% - 94.80%]	2.94% [2.78% - 3.10%]
		excluding exome	*7*	7.73% [2.67% - 28.41%]	6.88% [1.11% - 28.38%]	0.87% [0.03% - 2.08%]	*1*	28.30%	28.38%	0.08%
	Grade A variant lost	including exome	*5*	-	99.79% [99.29% - 99.94%]	-	*2*	-	99.99% [99.98% - 100.00%]	-
	Grade B variant lost	including exome	*2*	-	0.97% [0.68% - 1.27%]	-	*5*	-	5.56% [0.68% - 17.38%]	-
		excluding exome	*31*	-	1.24% [0.46% - 5.75%]	-	*37*	-	1.57% [0.46% - 6.20%]	-
	Grade C variant lost	from highCov	*66*	-	0.72% [0.41% - 2.71%]	-	*66*	-	0.72% [0.41% - 2.71%]	-
		from lowCov	*7*	-	0.52% [0.40% - 0.90%]	-	*7*	-	0.52% [0.40% - 0.90%]	-
		from exome	*2*	-	0.94% [0.84% - 1.04%]	-	*2*	-	0.94% [0.84% - 1.04%]	-
	Novel variant	Only present in Ion Torrent	*1*	2.75%	-	-	*3*	24.00% [19.30% - 31.00%]	-	-

Table S24 - Mixture analysis: Distribution of variants with freebayes and varscan

VCM	Variant description			PCP				TSS			
	Macro	Meso	Micro	*N*	Mean observed VL [Min - Max]	Within platform: Mean absolute difference in VLs [Min - Max]	Other platforms: Mean absolute difference in VLs [Min - Max]	*N*	Mean observed VL [Min - Max]	Within platform: Mean absolute difference in VLs [Min - Max]	Other platforms: Mean absolute difference in VLs [Min - Max]
freebayes	Found variants	Found variants	Major Grade A	*160*	92.54% [56.88% - 100.00%]	3.54% [0.06% - 18.12%]	3.81% [0.02% - 18.08%]	*165*	92.21% [56.80% - 100.00%]	3.46% [0.11% - 18.20%]	3.66% [0.07% - 18.16%]
			Major Grade B	*99*	64.69% [2.60% - 100.00%]	2.87% [0.10% - 22.99%]	3.38% [0.02% - 23.53%]	*80*	83.39% [11.80% - 100.00%]	3.19% [0.14% - 17.10%]	3.42% [0.02% - 17.33%]
			Major Grade C	*18*	91.91% [60.41% - 99.84%]	3.51% [0.57% - 14.59%]	3.36% [0.24% - 14.59%]	*15*	90.23% [57.60% - 99.60%]	3.68% [0.50% - 17.17%]	3.43% [0.20% - 17.40%]
			Minor Grade A	*78*	17.64% [2.99% - 52.77%]	4.50% [0.03% - 27.86%]	4.55% [0.01% - 28.07%]	*47*	25.41% [11.00% - 48.30%]	5.60% [0.00% - 23.50%]	5.64% [0.01% - 23.60%]
			Minor Grade B	*38*	14.75% [2.82% - 39.27%]	4.03% [0.15% - 14.29%]	4.04% [0.11% - 14.40%]	*19*	24.80% [11.90% - 37.50%]	6.17% [1.99% - 12.70%]	6.18% [2.05% - 12.62%]
			Minor Grade C	*6*	16.18% [5.04% - 35.66%]	5.48% [1.86% - 10.77%]	5.47% [1.82% - 10.66%]	*5*	23.26% [11.80% - 34.80%]	4.61% [1.85% - 9.92%]	4.63% [1.80% - 9.80%]
			Shared Grade A	*30*	99.82% [99.15% - 100.00%]	0.10% [0.00% - 0.36%]	0.15% [0.00% - 0.78%]	*30*	99.36% [98.60% - 100.00%]	0.28% [0.00% - 1.30%]	0.60% [0.02% - 1.33%]
			Shared Grade B	*75*	99.93% [99.62% - 100.00%]	0.08% [0.00% - 0.40%]	0.08% [0.00% - 0.35%]	*75*	99.68% [98.60% - 100.00%]	0.18% [0.00% - 0.69%]	0.31% [0.00% - 1.32%]
			Shared Grade C	*5*	99.70% [99.64% - 99.75%]	0.04% [0.00% - 0.08%]	3.51% [2.79% - 3.80%]	*10*	99.89% [99.60% - 100.00%]	0.06% [0.00% - 0.29%]	1.93% [0.06% - 4.08%]
		Mixture found variants	Major Grade B	*9*	4.08% [2.58% - 5.68%]	-	0.44% [0.02% - 1.67%]	*4*	12.60% [11.90% - 13.20%]	-	3.12% [0.83% - 4.00%]
			Shared Grade C	*13*	14.36% [3.95% - 99.80%]	-	5.77% [0.01% - 16.43%]	-	-	-	-
	Lost variants	Mixture lost variants	Major Grade B	*26*	-	19.63% [2.00% - 97.48%]	19.09% [0.84% - 98.91%]	-	-	-	-
			Major Grade C	*2*	-	82.37% [74.88% - 89.86%]	82.50% [75.00% - 90.00%]	-	-	-	-
			Minor Grade A	*82*	-	2.26% [0.99% - 5.00%]	2.26% [0.97% - 5.00%]	*113*	-	3.75% [0.99% - 10.00%]	3.76% [0.97% - 10.00%]
			Minor Grade B	*92*	-	1.93% [0.03% - 24.93%]	1.91% [0.01% - 24.98%]	*61*	-	3.61% [0.20% - 10.00%]	3.64% [0.22% - 10.00%]

VCM	Variant description			PCP				TSS			
	Macro	Meso	Micro	*N*	Mean observed VL [Min - Max]	Within platform: Mean absolute difference in VLs [Min - Max]	Other platforms: Mean absolute difference in VLs [Min - Max]	*N*	Mean observed VL [Min - Max]	Within platform: Mean absolute difference in VLs [Min - Max]	Other platforms: Mean absolute difference in VLs [Min - Max]
			Minor Grade C	*9*	-	2.66% [1.00% - 5.00%]	2.67% [1.00% - 5.00%]	*15*	-	4.12% [0.99% - 9.99%]	4.13% [1.00% - 10.00%]
		Old lost variants	Major Grade A	*5*	-	-	91.39% [74.99% - 98.99%]	-	-	-	-
			Major Grade B	*186*	-	-	0.89% [0.32% - 2.67%]	*236*	-	-	1.47% [0.32% - 16.50%]
			Major Grade C	*480*	-	-	0.57% [0.30% - 1.81%]	*480*	-	-	0.57% [0.30% - 1.81%]
			Minor Grade B	*190*	-	-	0.10% [0.00% - 1.44%]	*235*	-	-	0.16% [0.00% - 4.34%]
			Minor Grade C	*490*	-	-	0.14% [0.00% - 24.95%]	*485*	-	-	0.05% [0.00% - 0.46%]
			Shared Grade B	*5*	-	-	0.65% [0.56% - 0.83%]	*5*	-	-	0.65% [0.56% - 0.83%]
			Shared Grade C	*722*	-	-	1.47% [0.41% - 99.84%]	*730*	-	-	0.92% [0.41% - 4.57%]
	Novel variants	Found variants	Major novel	*2*	6.61% [6.45% - 6.77%]	0.68% [0.12% - 1.25%]	-	*20*	74.87% [22.30% - 99.80%]	3.94% [0.10% - 13.49%]	-
			Minor novel	*3*	3.57% [2.76% - 4.44%]	2.50% [2.33% - 2.65%]	-	*8*	24.80% [16.80% - 31.00%]	13.55% [5.90% - 24.07%]	-
			Shared novel	-	-	-	-	*10*	99.87% [99.60% - 100.00%]	0.25% [0.00% - 0.40%]	-
			Shared true novel	-	-	-	-	*4*	31.65% [28.00% - 35.10%]	12.10% [8.10% - 15.78%]	-
		Novel variants	Novel variant: mixture	*24*	4.85% [2.53% - 13.97%]	-	-	*9*	13.92% [10.60% - 21.10%]	-	-
	Primary novel variants	Mixture lost variants	Major novel	*13*	-	3.71% [1.89% - 8.36%]	-	*5*	-	28.33% [23.25% - 30.69%]	-
			Minor novel	*12*	-	0.22% [0.03% - 0.69%]	-	*17*	-	3.48% [0.19% - 9.99%]	-
			Shared true novel	-	-	-	-	*1*	-	19.35%	-

VCM	Variant description: Macro	Meso	Micro	PCP: N	PCP: Mean observed VL [Min - Max]	PCP: Within platform: Mean absolute difference in VLs [Min - Max]	PCP: Other platforms: Mean absolute difference in VLs [Min - Max]	TSS: N	TSS: Mean observed VL [Min - Max]	TSS: Within platform: Mean absolute difference in VLs [Min - Max]	TSS: Other platforms: Mean absolute difference in VLs [Min - Max]
varscan	Found variants	Found variants	Major Grade A	*250*	92.67% [56.88% - 100.00%]	3.42% [0.06% - 18.12%]	3.64% [0.01% - 18.08%]	*260*	92.10% [56.80% - 100.00%]	3.40% [0.10% - 18.20%]	3.53% [0.01% - 18.17%]
			Major Grade B	*39*	20.82% [2.60% - 94.34%]	1.72% [0.10% - 6.28%]	2.09% [0.31% - 6.15%]	*15*	43.18% [11.80% - 94.20%]	2.26% [0.14% - 6.29%]	2.26% [0.10% - 4.99%]
			Minor Grade A	*123*	17.24% [2.82% - 52.77%]	4.44% [0.03% - 27.86%]	4.48% [0.01% - 28.07%]	*73*	25.31% [11.00% - 48.30%]	5.73% [0.00% - 23.50%]	5.74% [0.01% - 23.60%]
			Minor Grade B	*8*	7.54% [3.27% - 26.01%]	1.90% [0.30% - 3.95%]	1.72% [0.12% - 4.18%]	*1*	26.30%	3.38%	2.60%
			Shared Grade A	*120*	99.92% [99.15% - 100.00%]	0.09% [0.00% - 0.40%]	0.19% [0.00% - 2.75%]	*125*	99.64% [98.60% - 100.00%]	0.20% [0.00% - 1.30%]	0.45% [0.00% - 2.73%]
		Mixture found variants	Major Grade A	*3*	98.06% [94.77% - 100.00%]	-	4.07% [1.71% - 5.41%]	-	-	-	-
			Major Grade B	*9*	4.08% [2.58% - 5.68%]	-	0.44% [0.02% - 1.67%]	*4*	12.60% [11.90% - 13.20%]	-	3.13% [0.84% - 4.01%]
			Minor Grade A	*2*	14.83% [8.84% - 20.83%]	-	2.65% [1.16% - 4.15%]	-	-	-	-
			Shared Grade A	*5*	100.00% [100.00% - 100.00%]	-	0.12% [0.07% - 0.22%]	-	-	-	-
	Lost variants	Mixture lost variants	Major Grade B	*21*	-	2.88% [2.00% - 3.58%]	1.89% [0.83% - 2.71%]	-	-	-	-
			Minor Grade A	*127*	-	2.39% [0.98% - 24.93%]	2.39% [0.97% - 24.90%]	*187*	-	3.86% [0.98% - 10.00%]	3.87% [0.97% - 10.00%]
			Minor Grade B	*52*	-	0.51% [0.03% - 4.63%]	0.51% [0.01% - 4.74%]	*14*	-	2.65% [0.20% - 9.17%]	2.78% [0.22% - 9.48%]
		Old lost variants	Major Grade A	*17*	-	-	90.67% [74.47% - 98.92%]	*10*	-	-	91.39% [74.99% - 99.00%]
			Major Grade B	*156*	-	-	0.94% [0.34% - 2.70%]	*206*	-	-	1.60% [0.34% - 16.52%]
			Major Grade C	*205*	-	-	0.55% [0.30% - 2.36%]	*205*	-	-	0.55% [0.30% - 2.36%]
			Minor Grade A	*18*	-	-	7.60% [1.00% - 24.98%]	*10*	-	-	8.60% [1.00% - 25.00%]

VCM	Variant description			PCP				TSS			
	Macro	Meso	Micro	*N*	Mean observed VL [Min - Max]	Within platform: Mean absolute difference in VLs [Min - Max]	Other platforms: Mean absolute difference in VLs [Min - Max]	*N*	Mean observed VL [Min - Max]	Within platform: Mean absolute difference in VLs [Min - Max]	Other platforms: Mean absolute difference in VLs [Min - Max]
			Minor Grade B	*165*	-	-	0.10% [0.00% - 1.44%]	*210*	-	-	0.18% [0.00% - 4.35%]
			Minor Grade C	*205*	-	-	0.05% [0.00% - 0.39%]	*205*	-	-	0.05% [0.00% - 0.39%]
			Shared Grade C	*170*	-	-	0.84% [0.42% - 2.70%]	*170*	-	-	0.84% [0.42% - 2.70%]
	Novel variants	Found variants	Major novel	-	-	-	-	*5*	28.48% [22.30% - 34.10%]	8.65% [1.03% - 13.49%]	-
			Minor novel	-	-	-	-	*5*	21.24% [16.80% - 26.00%]	18.07% [11.98% - 24.07%]	-
			Shared true novel	-	-	-	-	*4*	31.65% [28.00% - 35.10%]	12.10% [8.10% - 15.78%]	-
		Novel variants	Novel variant: mixture	*10*	3.39% [2.53% - 6.25%]	-	-	*9*	13.92% [10.60% - 21.10%]	-	-
	Primary novel variants	Mixture lost variants	Major novel	*5*	-	2.51% [2.06% - 2.72%]	-	*5*	-	28.33% [23.25% - 30.69%]	-
			Minor novel	*5*	-	0.24% [0.03% - 0.69%]	-	*5*	-	1.15% [0.19% - 3.10%]	-
			Shared true novel	-	-	-	-	*1*	-	19.35%	-

Table S25 - Ion Torrent sequencing of an independent set of 50 clinical samples: Overall look on differences between PCP and TSS

	PCP								TSS					
ID	Mappability >30 reads/base	Mappability >100 reads/base	Haplogroup	Haplogroup quality (%)	Contamination	Contamination level	Mean coverage	Number of variants	Haplogroup	Haplogroup quality (%)	Contamination	Contamination level	Mean coverage	Number of variants
1	99.95%	98.05%	H5a1c1a	96.27%	No	-	2417.09	18	H5a1c1a	96.27%	No	-	3282.00	16
2	99.88%	95.78%	H1	81.75%	No	-	1310.67	503	H1	90.08%	Yes	0.30%	1895.00	14
3	99.93%	96.92%	H	86.08%	No	-	1833.92	355	H7a1	92.25%	No	-	2717.00	14
4	99.54%	96.82%	H7	93.82%	No	-	1852.59	95	H7	93.82%	No	-	2524.00	10
5	99.05%	98.60%	V10b	93.92%	No	-	1820.96	37	V10b	93.92%	No	-	2479.00	17
6	99.95%	99.71%	H1+152	83.47%	No	-	1215.88	26	H1+152	83.47%	No	-	1823.00	14
7	99.90%	98.02%	T1a2*1	94.78%	No	-	2073.41	79	T1a2*1	95.32%	No	-	2866.00	37
8	99.82%	95.96%	L1b1a16	94.75%	No	-	1289.37	109	L1b1a16	94.65%	Yes	0.60%	2271.00	80
9	99.98%	99.95%	L3e1a2	97.54%	No	-	1393.70	55	L3e1a2	98.63%	No	-	1891.00	40
10	99.99%	99.94%	H80	96.54%	No	-	1575.96	15	H80	96.54%	No	-	2080.00	13
11	99.30%	97.91%	J1c2	94.04%	No	-	1048.60	74	J1c2	94.04%	No	-	1480.00	36
12	99.92%	98.94%	H1ak1	90.53%	No	-	1219.96	29	H1ak1	90.53%	No	-	1830.00	14
13	99.95%	98.79%	K1a4a1	94.44%	No	-	1216.21	41	K1a4a1	94.44%	No	-	1771.00	36
14	99.41%	98.58%	X2b+226	89.97%	No	-	1577.57	82	X2b	90.33%	No	-	2355.00	32
15	99.96%	99.60%	H1a	97.22%	No	-	1546.07	23	H1a	97.22%	No	-	2279.00	13
16	99.95%	99.20%	L1b1a12a	98.96%	No	-	1529.49	86	L1b1a12a	97.62%	No	-	2271.00	77
17	99.95%	99.81%	I2'3	95.13%	No	-	1687.41	35	I2'3	95.13%	Yes	0.40%	2659.00	32
18	99.23%	98.65%	L3e4a	98.57%	No	-	2125.65	32	L3e4a	98.57%	No	-	3039.00	29
19	99.91%	98.57%	L3b2a	95.89%	No	-	2367.50	59	L3b2a	95.89%	No	-	3411.00	38
20	99.96%	99.28%	I1a1	96.20%	No	-	2061.17	46	I1a1	96.22%	No	-	3108.00	37
21	99.89%	99.29%	H	82.33%	Yes	12.60%	2110.02	39	R0	88.61%	Yes	12.40%	3085.00	38
22	99.93%	99.23%	H1c	95.40%	No	-	1703.82	12	H1c	95.40%	Yes	0.70%	2616.00	11
23	99.90%	98.84%	L2a1c6	97.35%	No	-	1541.49	58	L2a1c6	97.35%	No	-	2324.00	57
24	99.65%	98.94%	J1b1a1	99.80%	No	-	2058.41	49	J1b1a1	98.20%	No	-	3192.00	37
25	99.68%	98.89%	H1e1a	100.00%	No	-	1636.08	16	H1e1a	100.00%	No	-	2627.00	12
26	99.95%	99.22%	I1a1	96.80%	No	-	1394.89	40	I1a1	95.01%	No	-	2168.00	37
27	99.96%	99.30%	H1	91.45%	No	-	1849.73	15	H1	91.45%	No	-	2705.00	13
28	98.95%	94.82%	H1+16189	100.00%	No	-	1158.02	31	H1+16189	100.00%	Yes	0.80%	2497.00	10
29	99.94%	98.86%	U6a2c	92.83%	No	-	1562.96	40	U6a2c	91.44%	No	-	2360.00	32

	PCP								TSS					
ID	Mappability >30 reads/base	Mappability >100 reads/base	Haplogroup	Haplogroup quality (%)	Contamination	Contamination level	Mean coverage	Number of variants	Haplogroup	Haplogroup quality (%)	Contamination	Contamination level	Mean coverage	Number of variants
30	99.96%	99.57%	H1	93.15%	No	-	1752.64	22	H1	93.15%	No	-	2584.00	11
31	99.97%	99.86%	U5b3	100.00%	No	-	2143.96	30	U5b3	100.00%	No	-	3176.00	26
32	99.97%	99.90%	H18	100.00%	No	-	2053.76	22	H18	100.00%	Yes	1.30%	3077.00	11
33	99.96%	99.89%	H1ba	89.65%	No	-	1702.02	30	H1	92.86%	No	-	2399.00	11
34	99.95%	99.51%	L2a1b1a	98.12%	No	-	1822.10	92	L2a1b1a	98.45%	No	-	3088.00	54
35	99.93%	97.59%	K1a+195	89.99%	No	-	2380.44	44	K1a3a	93.79%	No	-	3221.00	38
36	99.98%	99.89%	H5a4a1	91.99%	No	-	2239.32	38	H5a4a1	91.20%	No	-	3082.00	16
37	99.98%	99.89%	H1r	100.00%	Yes	3.30%	2871.44	34	H1r	100.00%	No	-	3906.00	10
38	99.97%	99.87%	U5a1a1	96.65%	No	-	2610.27	33	U5a1a1	96.65%	No	-	3479.00	29
39	99.96%	99.82%	J2b1a	96.74%	No	-	1864.20	45	J2b1a	96.74%	No	-	2513.00	33
40	99.31%	98.58%	L2b2a	98.39%	No	-	2917.67	85	L2b2a	99.33%	Yes	0.50%	3986.00	73
41	99.97%	99.20%	T1a5a	90.88%	No	-	1966.95	39	T1a5a	92.30%	No	-	2700.00	37
42	99.37%	98.18%	T2b3+151	94.86%	No	-	2410.06	47	T2b3+151	94.86%	Yes	0.40%	3254.00	38
43	99.98%	99.29%	K2a6	95.59%	No	-	2339.70	45	K2a6	98.22%	No	-	3212.00	32
44	99.99%	99.97%	H11a	94.57%	Yes	7.00%	2760.81	78	H11a	94.57%	No	-	3595.00	21
45	99.99%	99.98%	T1a1+@152	94.41%	No	-	2808.41	48	T1a1+@152	94.41%	Yes	0.60%	3717.00	37
46	99.99%	99.98%	J1c1b	96.39%	No	-	2777.32	47	J1c1b	96.39%	No	-	3683.00	31
47	99.94%	96.71%	H31	96.46%	No	-	1436.10	25	H31	96.46%	No	-	1983.00	13
48	99.97%	99.87%	W5	94.82%	No	-	2669.32	91	W5	94.82%	Yes	1.60%	3665.00	38
49	99.90%	97.68%	L2b1a	95.62%	No	-	1600.22	77	L2b1a	95.90%	No	-	2170.00	75
50	99.89%	97.37%	L3b1a1*	96.58%	No	-	1003.83	37	L3b1a1*	97.04%	No	-	1373.00	36

Table S26 - Ion Torrent sequencing of an independent set of 50 clinical samples: Discordant variants between PCP and TSS

Position	Mutation	Classification	*N*	Position blacklisted?	Mean VL	Mean normalized coverage	Coverage ratio	NUMT classification	Mean number of NUMTs	LCR	Distance to amplicon edge	HVS?	Poly-C?	*N* as homoplasmic variant in Helixmtdb	*N* as heteroplasmic variant in Helixmtdb
8649	8649C	PCP only	*25*		3.96%	0.30	1.88	pNUMT	3.00	No	2				
5821	5821A	PCP only	*21*		4.29%	2.15	1.18	NUMT (both)	16.00	No	22			*339*	*25*
4318	4318T	PCP only	*20*		4.20%	0.47	5.01	NUMT (both)	10.50	No	2			*7*	*0*
5840	5840T	PCP only	*20*		4.23%	2.18	1.10	NUMT (both)	21.00	No	18			*17*	*3*
10958	10958C	TSS only	*17*		21.66%	0.17	2.87	pNUMT	1.00	No	27				
6569	6569A	PCP only	*16*		4.21%	0.32	4.63	NUMT (both)	11.50	No	48				
1556	1556T	PCP only	*13*		4.12%	1.56	1.20	NUMT (both)	16.00	No	7			*1*	*2*
8152	8152A	PCP only	*13*		3.42%	0.76	19.95	NUMT (both)	4.50	No	35			*298*	*33*
3172	3172A	PCP only	*7*		3.20%	0.90	3.06	pNUMT	1.00	No	10			*30*	*0*
4253	4253A	PCP only	*6*		2.73%	0.55	7.87	NUMT (both)	1.00	No	37				
7957	7957T	PCP only	*6*		3.55%	0.57	3.21	NUMT (both)	1.50	No	37			*8*	*1*
7959	7959A	PCP only	*6*		3.23%	0.25	7.89	pNUMT	1.00	No	35				
8140	8140T	PCP only	*6*		3.05%	1.32	1.27	NUMT (both)	2.00	No	23			*12*	*0*
8943	8943T	PCP only	*6*		2.93%	0.71	1.56	NUMT (both)	5.50	No	51			*41*	*5*
9434	9434G	PCP only	*6*		3.05%	0.84	1.70	NUMT (both)	3.50	No	34			*1*	*1*
14715	14715C	PCP only	*6*		3.43%	0.39	21.15	NUMT (both)	4.50	No	1				
4456	4456T	PCP only	*5*		3.26%	0.86	1.44	NUMT (both)	8.50	No	31			*12*	*6*
6542	6542T	PCP only	*5*		4.10%	0.45	1.56	NUMT (both)	9.50	No	21			*23*	*0*
7146	7146G	PCP only	*5*		2.74%	1.09	1.86	NUMT (both)	7.50	No	33			*1174*	*282*
8065	8065A	PCP only	*5*		3.88%	2.29	1.12	NUMT (both)	7.00	No	41			*330*	*16*
11677	11677A	PCP only	*5*		2.86%	0.31	4.69	NUMT (both)	2.50	No	11			*4*	*0*
14429	14429A	PCP only	*5*		3.24%	0.26	10.45	NUMT (both)	4.50	No	5				*1*
204	204C	PCP only	*4*		51.45%	0.89	1.65	NUMT (both)	1.50	No	43	HVS-II		*11099*	*696*
498	498A	PCP only	*4*		3.55%	0.15	11.53	NUMT (both)	1.00	No	18	HVS-III			
1905	1905A	PCP only	*4*		3.45%	0.63	5.63	NUMT (both)	2.50	No	35				
2463	2463G	PCP only	*4*		99.48%	0.68	129.66	NUMT (both)	1.00	No	29				*1*
3913	3913T	PCP only	*4*		2.95%	0.45	7.22	NUMT (mtDNA-server)	1.00	No	18				
4048	4048A	PCP only	*4*		2.98%	0.66	1.38	NUMT (both)	1.00	No	44			*677*	*45*
4312	4312T	PCP only	*4*		3.08%	1.21	1.17	NUMT (both)	14.00	No	8			*419*	*7*

Position	Mutation	Classification	*N*	Position blacklisted?	Mean VL	Mean normalized coverage	Coverage ratio	NUMT classification	Mean number of NUMTs	LCR	Distance to amplicon edge	HVS?	Poly-C?	*N* as homoplasmic variant in Helixmtdb	*N* as heteroplasmic variant in Helixmtdb
5320	5320T	PCP only	*4*		3.63%	0.32	1.85	NUMT (both)	2.00	No	17			*13*	*0*
5752	5752G	PCP only	*4*		98.83%	0.42	54.48	NUMT (mtDNA-server)	2.00	No	36				
6410	6410T	PCP only	*4*		3.90%	0.81	1.53	NUMT (both)	15.50	No	3			*34*	*0*
7521	7521A	PCP only	*4*		3.35%	0.94	3.63	NUMT (both)	9.00	No	31			*2209*	*1190*
8021	8021G	PCP only	*4*		4.03%	2.25	1.28	NUMT (both)	6.00	No	16			*20*	*1*
8152	8152T	PCP only	*4*		3.68%	0.60	12.32	NUMT (both)	4.50	No	35			*3*	*0*
8167	8167C	PCP only	*4*		3.15%	1.39	1.24	NUMT (both)	3.00	No	50			*200*	*15*
8856	8856T	PCP only	*4*		3.13%	0.32	1.46	NUMT (both)	7.00	No	25			*4*	*0*
544	544A	PCP only	*3*		2.97%	0.13	14.95	NUMT (both)	1.00	No	1	HVS-III			*2*
1520	1520C	PCP only	*3*		3.20%	1.66	1.33	NUMT (both)	6.00	No	43			*54*	*2*
1536	1536G	PCP only	*3*		4.33%	1.64	1.18	NUMT (both)	6.00	No	27			*160*	*4*
1883	1883A	PCP only	*3*		3.23%	1.07	1.19	NUMT (both)	13.50	No	13				
1888	1888A	PCP only	*3*		3.97%	1.10	1.18	NUMT (both)	13.00	No	18			*18596*	*59*
1977	1977C	PCP only	*3*		3.27%	1.07	1.15	NUMT (both)	13.50	No	0			*89*	*5*
3897	3897A	PCP only	*3*		3.20%	0.35	12.89	NUMT (mtDNA-server)	14.00	No	2			*3*	*2*
4141	4141A	PCP only	*3*		2.90%	0.28	8.47	NUMT (both)	3.50	No	29				
4883	4883A	PCP only	*3*		3.70%	0.13	2.55	NUMT (both)	7.50	No	48				
5235	5235A	PCP only	*3*		3.87%	0.10	1.28	pNUMT	3.00	No	7				
5306	5306A	PCP only	*3*		3.03%	0.24	1.38	NUMT (both)	2.00	No	31				
5491	5491A	PCP only	*3*		3.17%	0.50	7.15	pNUMT	4.00	No	26				
5756	5756T	PCP only	*3*		3.17%	0.58	2.64	NUMT (mtDNA-server)	12.00	No	32				
6366	6366A	PCP only	*3*		4.13%	0.89	1.91	NUMT (both)	4.50	No	47			*176*	*12*
6383	6383A	PCP only	*3*		4.13%	0.88	1.85	NUMT (both)	6.00	No	30			*239*	*11*
7232	7232T	PCP only	*3*		3.70%	2.18	1.21	NUMT (both)	10.00	No	1			*3*	*2*
7316	7316A	PCP only	*3*		3.27%	1.70	1.29	NUMT (both)	6.00	No	21			*25*	*8*
8503	8503C	PCP only	*3*		4.00%	0.23	5.14	NUMT (both)	3.00	No	45			*237*	*7*
8701	8701G	PCP only	*3*		4.37%	0.08	4.10	NUMT (both)	2.50	No	43			*17154*	*47*
8718	8718G	PCP only	*3*		3.97%	0.08	2.60	NUMT (both)	3.00	No	51			*189*	*7*
9060	9060A	PCP only	*3*		3.27%	0.60	1.09	NUMT (both)	4.00	No	49				
9075	9075T	PCP only	*3*		3.73%	0.47	1.61	NUMT (both)	4.00	No	36			*13*	*0*

Position	Mutation	Classification	*N*	Position blacklisted?	Mean VL	Mean normalized coverage	Coverage ratio	NUMT classification	Mean number of NUMTs	LCR	Distance to amplicon edge	HVS?	Poly-C?	*N* as homoplasmic variant in Helixmtdb	*N* as heteroplasmic variant in Helixmtdb
9629	9629G	PCP only	*3*		2.83%	1.45	1.08	NUMT (both)	3.00	Yes	11			*49*	*5*
10635	10635T	PCP only	*3*		3.13%	0.23	1.59	NUMT (mtDNA-server)	1.00	No	19				
11657	11657T	PCP only	*3*		3.43%	0.71	1.80	pNUMT	1.00	No	31				
12684	12684A	PCP only	*3*		3.43%	1.46	1.36	NUMT (both)	10.00	No	22			*155*	*40*
12971	12971A	PCP only	*3*		2.60%	0.71	3.84	pNUMT	1.00	No	2				
13062	13062G	PCP only	*3*		3.63%	0.45	17.24	NUMT (both)	8.00	No	12			*11*	*44*
13095	13095C	PCP only	*3*		3.73%	1.04	2.58	NUMT (both)	2.50	No	11			*290*	*50*
13105	13105G	PCP only	*3*		3.63%	1.08	2.48	NUMT (both)	11.00	No	21			*4882*	*106*
14159	14159A	PCP only	*3*		3.43%	0.14	9.43	NUMT (both)	1.50	No	7				
14248	14248A	PCP only	*3*		3.93%	0.13	6.47	NUMT (both)	5.50	No	27				
14531	14531A	PCP only	*3*		4.77%	0.07	4.17	pNUMT	1.00	No	6				
15518	15518A	PCP only	*3*		3.23%	0.17	1.71	NUMT (mtDNA-server)	3.00	No	52			*1*	*0*
16183	16183C	PCP only	*3*		75.77%	0.11	7.72	pNUMT	5.00	Yes	38	HVS-I			
34	34T	PCP only	*2*		2.60%	1.31	1.25	NUMT (both)	11.00	No	19				
203	203A	PCP only	*2*		99.20%	0.43	5.45	NUMT (both)	1.50	Yes	44	HVS-II		*1201*	*21*
709	709A	PCP only	*2*		98.90%	1.13	1.43	NUMT (both)	11.00	No	0			*28750*	*168*
710	710C	PCP only	*2*		99.35%	1.04	1.42	NUMT (both)	16.50	No	1			*998*	*5*
1120	1120T	PCP only	*2*		2.85%	0.64	3.13	NUMT (both)	10.50	No	2			*67*	*3*
1462	1462A	PCP only	*2*		6.65%	0.62	1.04	NUMT (both)	14.50	No	1			*584*	*12*
1476	1476A	PCP only	*2*		2.55%	1.57	1.29	NUMT (both)	7.50	No	2				
1488	1488A	PCP only	*2*		2.70%	1.42	1.51	NUMT (both)	7.50	No	14				
1518	1518T	PCP only	*2*		2.80%	1.60	1.23	NUMT (both)	7.50	No	44			*18*	*1*
1534	1534A	PCP only	*2*		2.85%	1.36	1.22	pNUMT	11.00	No	29				
1944	1944T	PCP only	*2*		3.65%	1.11	1.20	NUMT (both)	2.50	No	33			*1*	*0*
2219	2219A	PCP only	*2*		2.75%	0.21	2.00	NUMT (mtDNA-server)	3.00	No	20			*1*	*0*
2772	2772A	PCP only	*2*		2.60%	0.87	1.33	NUMT (mtDNA-server)	2.00	No	0			*5*	*0*
3914	3914T	PCP only	*2*		2.75%	0.71	1.55	Non-NUMT		No	19				
4463	4463A	PCP only	*2*		3.10%	0.64	1.11	NUMT (both)	5.00	No	24				
4479	4479A	PCP only	*2*		3.50%	0.57	2.50	NUMT (both)	2.00	No	8				
4856	4856C	PCP only	*2*		2.85%	0.38	2.57	NUMT (both)	2.00	No	21			*204*	*6*

Position	Mutation	Classification	*N*	Position blacklisted?	Mean VL	Mean normalized coverage	Coverage ratio	NUMT classification	Mean number of NUMTs	LCR	Distance to amplicon edge	HVS?	Poly-C?	*N* as homoplasmic variant in Helixmtdb	*N* as heteroplasmic variant in Helixmtdb
5111	5111T	PCP only	*2*		2.90%	0.78	1.68	NUMT (both)	1.50	No	16			*19*	*0*
5147	5147A	PCP only	*2*		3.05%	0.27	2.27	NUMT (both)	4.50	No	9			*8213*	*664*
5580	5580C	PCP only	*2*		2.60%	1.06	1.13	NUMT (both)	4.50	No	6			*383*	*29*
6173	6173A	PCP only	*2*		2.70%	0.83	4.27	NUMT (both)	2.50	No	4				
6381	6381T	PCP only	*2*		3.00%	0.29	6.50	NUMT (both)	3.00	No	32				
6424	6424A	PCP only	*2*		3.80%	0.30	8.86	NUMT (both)	3.00	No	0				
6641	6641C	PCP only	*2*		2.60%	2.09	1.23	NUMT (both)	8.00	No	4			*42*	*15*
7256	7256T	PCP only	*2*		3.35%	2.08	1.12	NUMT (both)	11.00	No	23			*4195*	*151*
7705	7705C	PCP only	*2*		3.50%	1.21	1.29	NUMT (both)	3.50	No	48			*1158*	*58*
8276	8276A	PCP only	*2*		2.70%	0.53	20.71	NUMT (both)	2.00	No	48			*10*	*1*
8455	8455T	PCP only	*2*		3.70%	0.23	7.46	NUMT (both)	2.00	No	15			*109*	*2*
8545	8545A	PCP only	*2*		3.65%	0.26	13.28	NUMT (both)	4.00	No	3			*138*	*16*
8677	8677C	PCP only	*2*		3.20%	0.09	4.50	NUMT (both)	4.50	No	19				
9055	9055T	PCP only	*2*		2.90%	0.45	2.06	pNUMT	2.00	No	44				*1*
9478	9478A	PCP only	*2*		4.40%	1.04	7.46	NUMT (both)	3.50	No	1				
9573	9573A	PCP only	*2*		2.90%	0.20	2.16	pNUMT	1.00	No	5				
10196	10196A	PCP only	*2*		3.10%	0.61	21.24	NUMT (mtDNA-server)	1.00	No	34				
10385	10385G	PCP only	*2*		99.30%	0.84	95.78	NUMT (mtDNA-server)	1.00	No	8			*4*	*1*
11063	11063A	PCP only	*2*		2.85%	0.46	2.23	pNUMT	1.00	No	35				
11093	11093T	PCP only	*2*		3.40%	0.24	1.07	pNUMT	1.00	No	5				
11237	11237A	PCP only	*2*		2.85%	0.18	2.39	pNUMT	3.00	No	52				
11708	11708G	PCP only	*2*		2.70%	1.21	1.74	NUMT (both)	2.00	No	9			*6*	*3*
11767	11767T	PCP only	*2*		2.60%	1.19	1.55	NUMT (both)	5.50	No	4			*9*	*1*
12705	12705T	PCP only	*2*		3.75%	1.49	1.43	NUMT (both)	6.50	Yes	43			*35323*	*91*
12838	12838T	PCP only	*2*		3.10%	1.29	1.20	Non-NUMT		No	30				
13359	13359A	PCP only	*2*		3.05%	0.76	1.21	NUMT (both)	14.50	No	33			*346*	*26*
13368	13368A	PCP only	*2*		3.40%	0.74	1.15	NUMT (both)	10.00	No	42			*17444*	*42*
13386	13386C	PCP only	*2*		3.10%	0.77	1.16	NUMT (both)	13.50	No	56			*81*	*4*
13440	13440T	PCP only	*2*		3.05%	0.74	1.16	NUMT (both)	4.50	No	2			*44*	*0*
13766	13766A	PCP only	*2*		4.85%	0.06	1.44	NUMT (both)	1.00	No	12			*5*	*0*

Position	Mutation	Classification	*N*	Position blacklisted?	Mean VL	Mean normalized coverage	Coverage ratio	NUMT classification	Mean number of NUMTs	LCR	Distance to amplicon edge	HVS?	Poly-C?	*N* as homoplasmic variant in Helixmtdb	*N* as heteroplasmic variant in Helixmtdb
14493	14493A	PCP only	*2*		3.70%	0.20	18.96	NUMT (both)	4.50	No	44				
14771	14771T	PCP only	*2*		4.10%	0.13	13.45	NUMT (both)	1.50	No	55			*8*	*0*
14777	14777C	TSS only	*2*		12.85%	0.28	2.15	NUMT (both)	1.50	No	59				*1*
16131	16131C	PCP only	*2*		2.70%	2.11	1.15	pNUMT	2.00	No	0	HVS-I		*60*	*18*
16380	16380A	PCP only	*2*		2.90%	0.32	12.35	pNUMT	6.00	No	39				

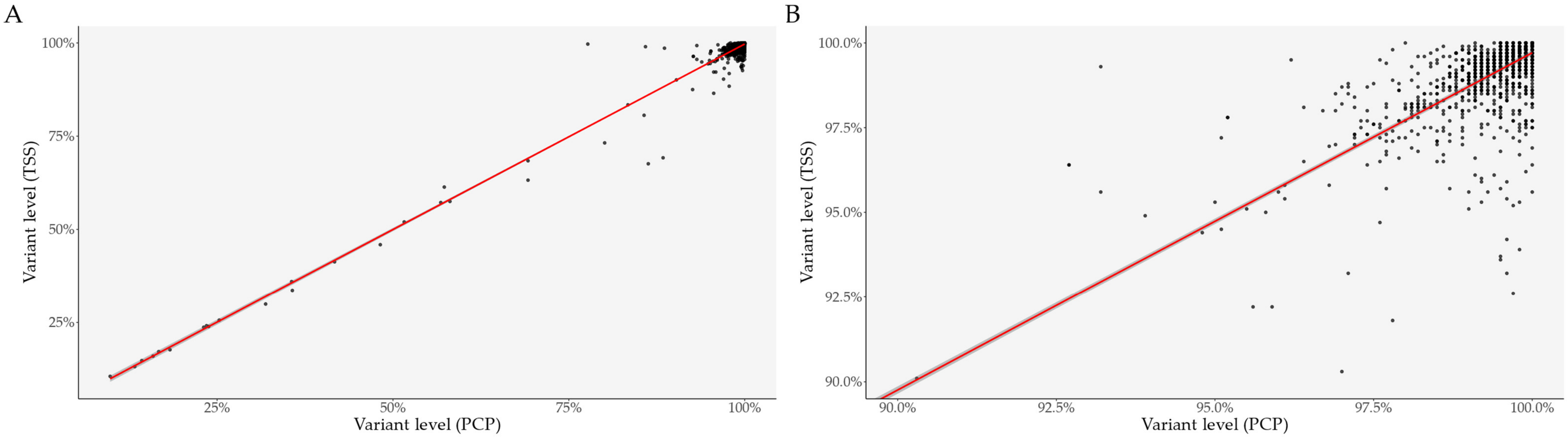

Figure S5 - Ion Torrent sequencing of an independent set of 50 clinical samples: Linear regression model between the variant level in PCP and the variant level in TSS, for variants found in both PCP and TSS.

(A) Linear regression model between the variant level in PCP and the variant level in TSS for all variants found in both PCP and TSS, (**B**) Close-up of Figure S5A, focusing on homoplasmic variants.
